# Hallmark-guided subtypes of hepatocellular carcinoma for the identification of immune-related gene classifiers in the prediction of prognosis, treatment efficacy, and drug candidates

**DOI:** 10.3389/fimmu.2022.958161

**Published:** 2022-08-10

**Authors:** Chengbin Guo, Yuqin Tang, Zhao Yang, Gen Li, Yongqiang Zhang

**Affiliations:** ^1^ Guangzhou Institute of Pediatrics, Guangzhou Women and Children’s Medical Center, Guangzhou Medical University, Guangzhou, China; ^2^ State Key Laboratory of Southwestern Chinese Medicine Resources, School of Basic Medical Sciences, Chengdu University of Traditional Chinese Medicine, Chengdu, China; ^3^ West China School of Medicine, West China Hospital, Sichuan University, Chengdu, China; ^4^ Wuhan Institute of Virology, Center for Biosafety Mega-Science, Chinese Academy of Sciences, Wuhan, China

**Keywords:** hepatocellular carcinoma, hallmark gene sets and molecule subtypes, immunotherapy efficacy, immune infiltration, prognosis, molecular docking

## Abstract

Hepatocellular carcinoma (HCC), accounting for ~90% of all primary liver cancer, is a prevalent malignancy worldwide. The intratumor heterogeneity of its causative etiology, histology, molecular landscape, and immune phenotype makes it difficult to precisely recognize individuals with high mortality risk or tumor-intrinsic treatment resistance, especially immunotherapy. Herein, we comprehensively evaluated the activities of cancer hallmark gene sets and their correlations with the prognosis of HCC patients using gene set variation analysis (GSVA) and identified two HCC subtypes with distinct prognostic outcomes. Based on these subtypes, seven immune-related genes (TMPRSS6, SPP1, S100A9, EPO, BIRC5, PLXNA1, and CDK4) were used to construct a novel prognostic gene signature [hallmark-guided subtypes-based immunologic signature (HGSIS)] *via* multiple statistical approaches. The HGSIS-integrated nomogram suggested an enhanced predictive performance. Interestingly, oncogenic hallmark pathways were significantly enriched in the high-risk group and positively associated with the risk score. Distinct mutational landscapes and immune profiles were observed between different risk groups. Moreover, immunophenoscore (IPS) and tumor immune dysfunction and exclusion (TIDE) analysis showed different sensitivities of HGSIS risk groups for immune therapy efficacy, and the pRRophetic algorithm indicated distinguishable responses for targeted/chemotherapies in different groups. KIF2C was picked out as the key target concerning HGSIS, and the top 10 small molecules were predicted to bind to the active site of KIF2C *via* molecular docking, which might be further used for candidate drug discovery of HCC. Taken together, our study offers novel insights for clinically significant subtype recognition, and the proposed signature may be a helpful guide for clinicians to improve the treatment regimens.

## Introduction

Liver cancer is the fourth leading cause of cancer-related death and hepatocellular carcinoma (HCC) is the most prevalent type of liver cancer (1). The infection of hepatitis B virus (HBV) and hepatitis C virus (HCV) is the main etiological risk factors for HCC, although non-alcoholic steatohepatitis (NASH) associated with metabolic syndrome or diabetes mellitus is the fastest-growing cause of HCC, especially in the West (2). HCC is a highly heterogeneous disease, which arises in a background of long-time chronic liver diseases in most cases (3). HCC heterogeneity is constituted by multiple features including genomic instability, molecular and signal transduction network disorders, and microenvironment discrepancies, contributing to the main reason for the ineffectiveness of traditional treatment (4–6). Likewise, the intralesional, interlesional, and intertumoral heterogeneity of HCC is challenging the prognostic prediction and personalized therapy development for HCC patients (7). Thus, identification of HCC subtypes with clinical significance and novel prognostic biomarkers or signatures are urgently needed for improved risk stratification and personalized treatment in HCC patients.

Exploring molecular alterations and signaling pathways related to cancer hallmarks is critical for classifying HCC subtypes to devise personalized treatments. While traditional experimental approaches that focus on one signaling pathway or a molecule could provide insight into the understanding of cancer initiation or progression, they are not suitable to develop a valid standard of HCC classification for further risk assessment. Meanwhile, gene set-based approaches have attracted considerable attention for HCC risk stratification recently; for example, ferroptosis-related genes, hypoxia-related genes, and lipid metabolism-related genes have been investigated to develop prognostic gene signatures in HCC (8–10). However, systematic exploration of cancer hallmark-related multiple gene sets to define HCC subtypes with appealing implications for the prediction of prognosis, treatment responses, and candidate drugs is still limited.

Recent studies have found that the tumor immune microenvironment (TIME) has a significant impact on the occurrence and development of tumors (11, 12). In HCC, cancer immunotherapy is developing rapidly since encouraging clinical outcomes have been obtained with monoclonal antibodies (mAbs), which target immune checkpoints to reverse the inactivation of T cells to eliminate tumor cells. Hopefully, for patients with advanced HCC, nivolumab, the PD1 inhibitor, was approved in the United States (13). Tremelimumab, an anti-CTLA4 immune checkpoint inhibitor (ICI), made exciting progress in a clinical trial (14). The combination of the anti-PD1 antibody atezolizumab and the VEGF-neutralizing antibody bevacizumab is exceedingly promising as a first-line drug for the treatment of HCC (15). However, immune cells constitute the trickiest component of the tumor microenvironment (TME) in HCC, of which the heterogeneity poses a significant challenge for the classification of HCC, leading to the uncertainty of prognosis (16).

**Figure 1:**
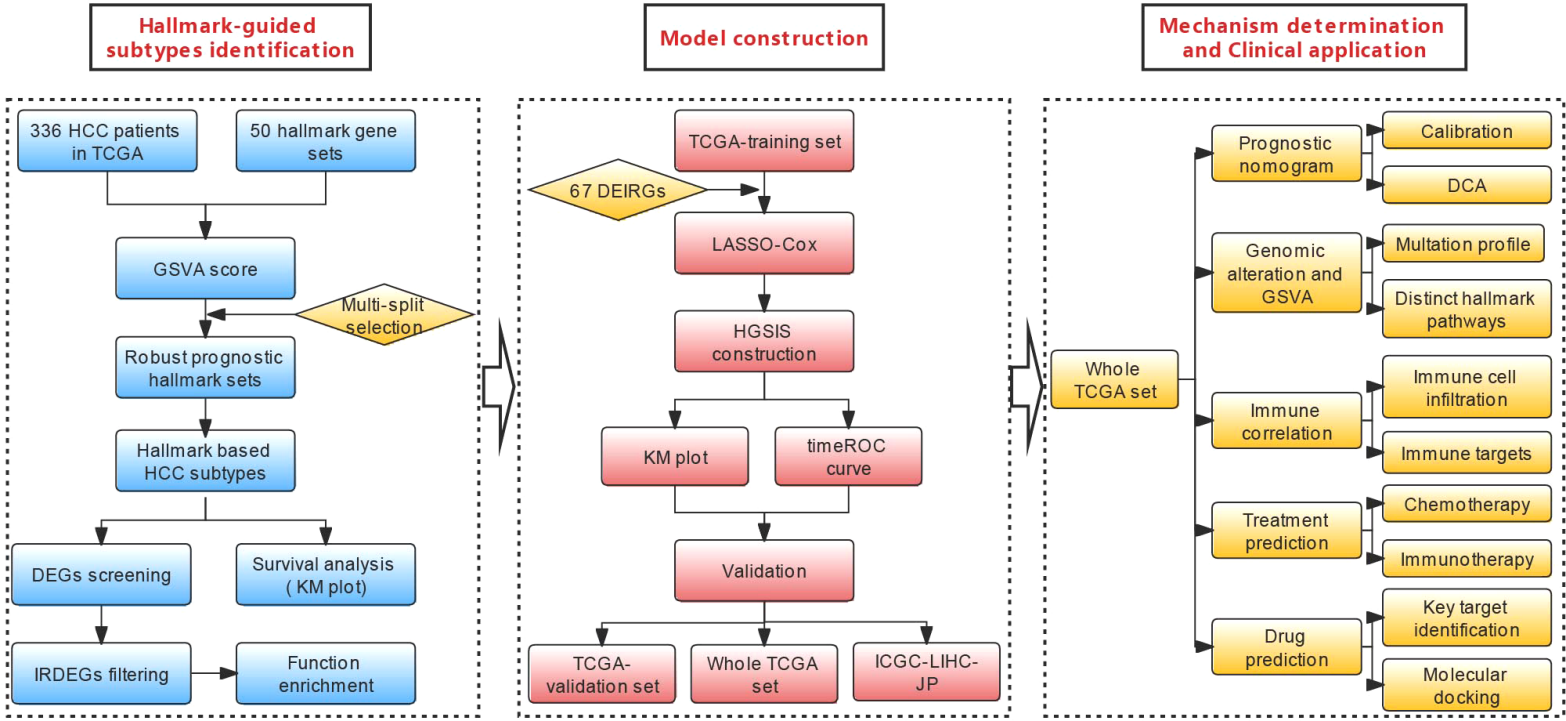
Flowchart of the current study.

Gene set variation analysis (GSVA) is a state-of-the-art framework to generate sample-level pathway scores in an unsupervised manner from gene expression profiles, which represents the starting point to develop pathway-centric models of biology and provides increased power than other sample-wise enrichment approaches to evaluate the variation of pathway activity. Compared with the popular gene set enrichment analysis (GSEA) method, GSVA is a more convenient algorithm without having to pre-define the classes of a given sample population, and it provides greater biological interpretability (17). In the present study, the GSVA scores of 50 hallmark gene sets from the molecular signature database (MSigdb) (18, 19) were computed using the TCGA-LIHC dataset, and robust prognostic hallmark gene sets were comprehensively screened and used to generate two HCC subtypes with divergent survival outcomes. Based on the two subtypes, a seven-gene immunologic signature that was named HGSIS for predicting the prognosis of HCC was established and validated with multiple statistical approaches. Distinct TIME profiles and mutational landscapes regarding HGSIS were characterized, and the significant association between HGSIS and immunotherapy efficacy was unraveled. Notably, we predicted candidate drugs that might bind to the crucial target of HGSIS with molecular docking. The flowchart of the study is shown in **Figure 1**.

## Materials and methods

### Patient information and data collection

Gene expression data of HCC patients were downloaded from The Cancer Genome Atlas (TCGA) and International Cancer Genome Consortium (ICGC) databases. Those patients with incomplete overall survival (OS) information and with an OS time of <30 days were excluded as reported before (20, 21). The clinicopathological features and other types of survival outcomes including progression-free survival (PFS), disease-free survival (DFS), and disease-specific survival (DSS) were also collected. The transcriptomic stemness index mRNAsi evaluating the degree of cancer stemness for each of all the HCC patients from the TCGA dataset was computed with the OCLR-based algorithm (22). Consequently, 336 samples from TCGA (the whole TCGA cohort) and 238 samples from the ICGC (ICGC-LIHC-JP) were included in the study. The whole TCGA cohort was randomly divided into the training dataset (*n* = 222) and validation dataset (*n* = 114) with an approximate ratio of 2:1. Moreover, the whole TCGA cohort and the ICGC-LIHC-JP cohort further served as the internal and external validation sets. The patient population and the clinicopathological characteristics are summarized in **Supplementary Table 1**. The normalized RNA sequencing profiles were retrieved and preprocessed as previously reported (20, 23, 24). For the analysis of somatic mutation information, we gathered the available mutation annotation format (MAF) file from the TCGA data portal (http://tcga-data.nci.nih.gov/tcga/) using the “maftools” package (25). Additionally, 1,118 unique immune-related genes (IRGs) were achieved from the Immunology Database and Analysis Portal (ImmPort) database (https://www.immport.org/home) (26).

### Gene set variation analysis and consensus clustering

The relative enrichment scores of the 50 cancer hallmark gene sets from Msidb (h.all.v7.1.symbols) (19) for the whole TCGA cohort, which were used to estimate the activities of these cancer hallmark pathways, were computed by the GSVA algorithm using the “GSVA” package (17). Kaplan–Meier analysis with log-rank test was utilized to examine the associations between each gene set and the OS. To increase the robustness of the prognostic gene sets, we adopted the “multi-split” strategy with 100 randomized subsamples as we reported before (24), and only those that repeatedly showed significance in all 100 times were considered as prognostic gene sets (**Supplementary Figure 1**). Based on the prognostic gene sets, unsupervised clustering was applied for all the HCC patients from TCGA with the “ConsensusClusterPlus” package (27) to distinguish different molecular patterns with divergent OS outcomes. This process was performed with 1,000 iterations by sampling 80% of all the data for each iteration to ensure clustering stability. The optimal clustering number was comprehensively determined by the item-consensus plots, the consensus heatmap, and the change in the area under the cumulative distribution function (CDF) curves, which was further confirmed by the proportion of ambiguous clustering (PAC) algorithm (28, 29). Two hallmark-guided subtypes with distinct OS outcomes were recognized and visualized with principal component analysis (PCA) plots. Additionally, Kaplan–Meier plots were depicted to evaluate the prognosis of patients in different Hallmark-guided subtype groups.

### Screening of the immune-related DEGs (IRDEGs) between HCC subtypes

Differentially expressed genes (DEGs) between the two hallmark-guided subtypes were screened by the “limma” package with the criteria of adjusted *p*-values <0.01 and |logFC| >1 (30, 31). A volcano plot was drawn to show these DEGs using the “ggplot2” package (https://cran.r-project.org/web/packages/ggplot2), followed by the identification of IRDEGs with a Venn plot. The “pheatmap” package was utilized to show the IRDEGs’ expression patterns between the two HCC subtypes. As previously described, Gene Ontology (GO) enrichment and Kyoto Encyclopedia of Genes and Genomes (KEGG) analysis for these IRDEGs were conducted *via* the “clusterProfiler” package with an adjusted *p* < 0.05 (20, 23, 32).

### Signature establishment and evaluation

The aforementioned IRDEGs were next subjected to the comprehensive feature selection, followed by the construction of the HGSIS signature on the training set, whose effectiveness and performance were evaluated on the training and validation datasets. Specifically, Univariate Cox (UniCox) proportional hazards regression analysis was used to pick out candidate IRDEGs with prognostic significance according to the criteria of *p* < 0.05. The least absolute shrinkage and selection operator (LASSO) regression algorithm was then applied by the “glmnet” package to find out the best subset of prognostic genes (33). We chose the significant IRDEGs that repeatedly appeared more than 50 times from 100 models to develop the scoring system HGSIS: Risk score = Σ(coef (*β*)*EXP*_β_*), where *β* stands for each selected IRDEG. All patients were classified into the high- and low-risk groups using the median risk score of the training set.

To assess the reliability of HGSIS, Kaplan–Meier curves were depicted for the TCGA training set, the TCGA validation set, the whole TCGA cohort, and the external validation set to compare the OS of different risk groups *via* the “survival” package. Time-dependent receiver operating characteristic (tROC) curves were also drawn to evaluate the predictive performance of HGSIS. In addition, we compared the area under the curve (AUC) values of the 3-year and 5-year tROC curves between HGSIS and other published HCC signatures as well as popular biomarkers for immunotherapy, i.e., a TP53-associated gene signature by Long et al. (“Long signature”) (34) and two immune-related gene signatures (“Dai signature” and “Wang signature”) (35, 36), TMB, and PD1.

### Correlation of HGSIS with clinicopathological features

The relationship between HGSIS and clinicopathological parameters was examined using nonparametric tests and visualized by the “ggplot2” package. The correlation between HGSIS and mRNAsi was measured using the Pearson correlation test *via* the “ggstatsplot” package. Stratified survival analysis was carried out for selected clinicopathological factors such as age, gender, and BMI to further validate the additional prognostic value of the HGSIS model. Then, univariate and multivariate regression analyses were conducted to verify the independent prognostic value of HGSIS in HCC. Based on the univariate analysis, an HGSIS-integrated nomogram was created by the “rms” package to quantitatively predict the OS probability, whose predictive accuracy was evaluated by calibration plots. The concordance index was further used to assess its performance. Moreover, decision curve analysis (DCA) was used to explore the potential clinical benefit of HGSIS as described (20). Additionally, Kaplan–Meier analysis with a log-rank test was applied to the output of the nomogram-based classifier for the whole TCGA cohort to further compare the differences in OS, DFS, PFS, and DSS between different risk classes.

### Genomic alterations and hallmark pathway analysis

Genomic mutations have been reported to be relevant to immunity and immunotherapy (20, 37–39); thus, we explored the somatic mutation analysis for the HGSIS high- and low-risk groups. The “maftools” R package was used to depict the waterfall plots showing the mutation landscapes of different risk groups of the whole TCGA cohort. Mutation types and frequencies of the most commonly mutated genes in each risk group were manifested. TMB values were computed with non-synonymous mutations as described previously to reveal the total mutation numbers of HCC patients (40, 41). Meanwhile, a linear model was employed to compare the GSVA scores of the 50 cancer hallmark gene sets between HGSIS risk groups to uncover the relative activities of these pathways in terms of HGSIS (42). Those with an adjusted *p*-value of < 0.01 were defined as significant gene sets and Kaplan–Meier analysis was then used to verify the prognostic value of typical oncogenic hallmark pathways.

### TIME patterns and immunological targets of HGSIS

For the estimation of TIME patterns regarding HGSIS, ssGSEA, a deconvolution algorithm implemented in the GSVA package (17, 43), was utilized to quantify the compositions of 30 types of TME cells, namely, 28 adaptive and innate immune cell types (44) and 2 stromal components (fibroblasts and endothelial cells) (45) based on the transcriptional data of the whole TCGA cohort. The ssGSEA scores representing the abundance of these TIME cells were next compared between different HGSIS risk groups, and the Spearman correlation analysis was performed to investigate the relationship between the HGSIS risk score and each TIME cell type. The prognostic values of these TIME cells were examined by Kaplan–Meier survival analysis. Furthermore, we contrasted the expression levels of 50 immunological targets that were classified into several groups such as receptors, ligands, and co-inhibitors (20, 46–48) to determine the intrinsic immune escape regarding HGSIS groups.

### Prediction of therapeutic responses

Based on the HCC patients’ data from TCGA, we predicted the putative sensitivities of HGSIS in immunotherapy and targeted/chemotherapies. For immunotherapy responses, we used the immunophenoscore (IPS) (44), which is calculated *via* machine learning and could be derived from The Cancer Immunome Atlas (TCIA) (https://tcia.at/home) to represent tumor immunogenicity of HCC patients. Moreover, the tumor immune dysfunction and exclusion (TIDE) score is a framework that was developed to infer the possible influences on survival and responses to immunotherapy. Two primary mechanisms (T-cell exclusion and T-cell dysfunction) of tumor immune evasion were integrated by the TIDE algorithm (http://tide.dfci.harvard.edu/) (49) with gene expression profiles of large cohorts to determine the clinical response to immunotherapy of HCC patients. The differences of IPS and TIDE scores between different groups were compared by the Wilcoxon test, and a lower TIDE score and a higher IPS indicate better sensitivities to immunotherapy. Furthermore, the half-maximal inhibitory concentration (IC_50_) values of 138 drugs were estimated by the “pRRophetic package” (50) and further normally transformed to evaluate the predictive capacity of HGSIS for the responses to targeted/chemotherapies.

### PPI network construction and key target identification

The limma package (31) was adopted to screen the DEGs between the HGSIS high- and low-risk groups using the whole TCGA cohort, and an adjusted *p*-value <0.01 and |logFC| ≥1.5 was set as the cutoff. The DEGs were then uploaded to the STRING database (version 11.5), an online database for the investigation of interactive relationships among proteins, to build a PPI network with a combined confidence score of ≥0.7. The STRING-based PPI network was next imported into Cytoscape (51) (version 3.8.2) for visualization. Furthermore, the MCODE plugin (52) was applied for cluster analysis and seed nodes identification, which were considered the key targets.

### Molecular docking

The process of molecular docking was completed with Glide of Schrodinger as previously reported (20). Specifically, the crystal structure of the key target of HGSIS was derived from the RCSB PDB database (www.rcsb.org/), followed by the recognition of its active site using the DeepSite tool (53) from the PlayMolecule platform (https://www.playmolecule.com/). The protein docking structure was prepared by the Protein Preparation Wizard in the Maestro 11.6 version of the Schrödinger suite. Additionally, 111,178 compounds’ structures involved in the in-man subset were downloaded from the ZINC 15 database (https://zinc15.docking.org). The virtual screening was conducted with the Glide Virtual Screening Workflow module integrated in the Schrödinger suite, the three main steps of which were applied to screen the candidates of KIF2C active affinity ligands. The first step was the high-throughput virtual screening (HTVS) mode starting with the 111,178 compounds, and subsequently, compounds with the top 10% of HTVS score were measured by the SP (Standard Precision) docking method. The third step was XP (Extra Precision) for the calculation of the top 10% SP docking score ranked compounds. OPLS-2005 force field was used during ligand–protein docking analysis to estimate the binding affinity.

### Statistical analysis

The R package “survival” was utilized to pick out the significant hallmark gene sets, IRDEGs, and clinicopathological factors for OS, together with the hazard ratios (HRs) and 95% confidence intervals (CIs). Kaplan–Meier analysis with a log-rank test was used to analyze the differences between two subgroups of categorical variables for the OS, DFS, PFS, and DSS of HCC patients. The best cutoff for Kaplan–Meier survival analysis was determined by using the “survminer” package. Multivariate analysis was used to identify independent prognostic indicators. The package “timeROC” was used to depict the tROC curves to assess the predictive ability of HGSIS for OS. The comparison of survival rates between different risk groups was completed using the Pearson Chi-square test. Wilcoxon test was used to compare the distribution of continuous data for three or more groups and Kruskal–Wallis test was used to determine the statistical difference of that for two groups. Correlations between two quantitative variables were explored by Pearson’s correlation test. All the above statistical analyses were conducted by the R software (version 3.6.1). Unless otherwise noted, *p* < 0.05 was considered statistically significant.

## Results

### Hallmark-guided recognition of HCC subtypes with prognostic significance

**Figure 2:**
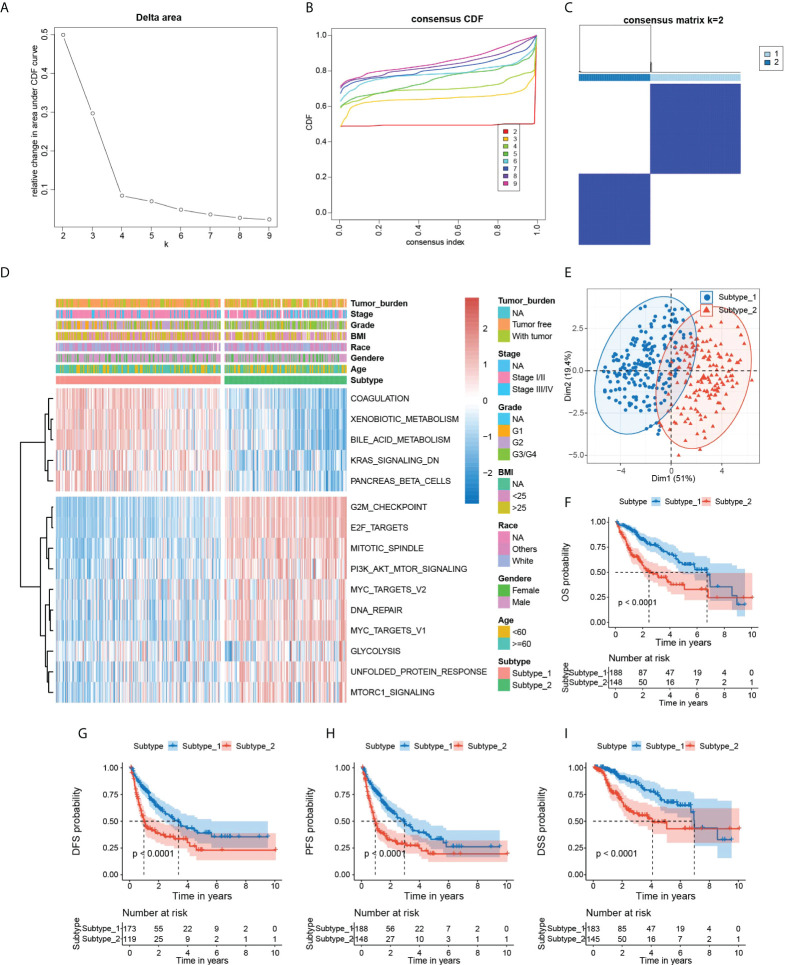
Identification of hallmark-based HCC subtypes**. (A)** The corresponding relative change in area under the cumulative distribution function **(C, D, F)** curves and the optimal number of cluster(*k*) was 2. **(B)** Consensus clustering CDF for *k* = 2 to 9. **(C)** Heatmap of sample clustering at consensus *k* = 2. **(D)** Heatmap showing the GSVA score of 15 hallmark gene sets, tumor burden, stage, grade BMI, race, gender, and age in two subtypes of HCC. **(E)** PCA plot visualizing the two HCC subtypes with 15 hallmark gene sets. **(F–I)** Kaplan–Meier survival plots of subtype 1 and subtype 2 for OS, DFS, PFS, and DSS. OS, overall survival. DFS, disease-free survival. PFS, progression-free survival. DSS, disease-specific survival. PCA, principal component analysis. GSVA, Gene set variation analysis.

Hallmark gene sets from MSigDB are coherently expressed signatures representing well-defined biological states or processes; thus, it is reasonable to identify specific hallmark-based HCC subtypes with distinct prognoses. Motivated by this rationale, we obtained 336 HCC patients’ mRNA expression matrix with corresponding clinical information from the TCGA database, followed by the computation of the enrichment scores for all 50 hallmark gene sets of each sample by GSVA. Next, we screened the robust hallmark gene sets significantly correlated to the prognosis of HCC patients with the “multi-split” strategy (**Supplementary Figure 1**). As a result, 15 hallmark gene sets were consistently significant 100 times in 100 subsamples, which were considered as prognostic hallmark gene sets. Based on the 15 hallmark gene sets, we performed the unsupervised clustering analysis for subtype classification. Our results showed that the optimal number of clusters was 2, which generated the greatest increase in the area under the CDF curves, and it was further validated by the PAC algorithm (**Figures 2A–C** and **Supplementary Figure 2**). Thus, we further classified all HCC patients into two distinct subtypes (**Figures 2D, E**). Notably, Kaplan–Meier analysis found that the patients in subtype 2 had a shorter survival time than the patients in subtype 1 (**Figures 2F–I**).

### Construction of HGSIS

**Figure 3:**
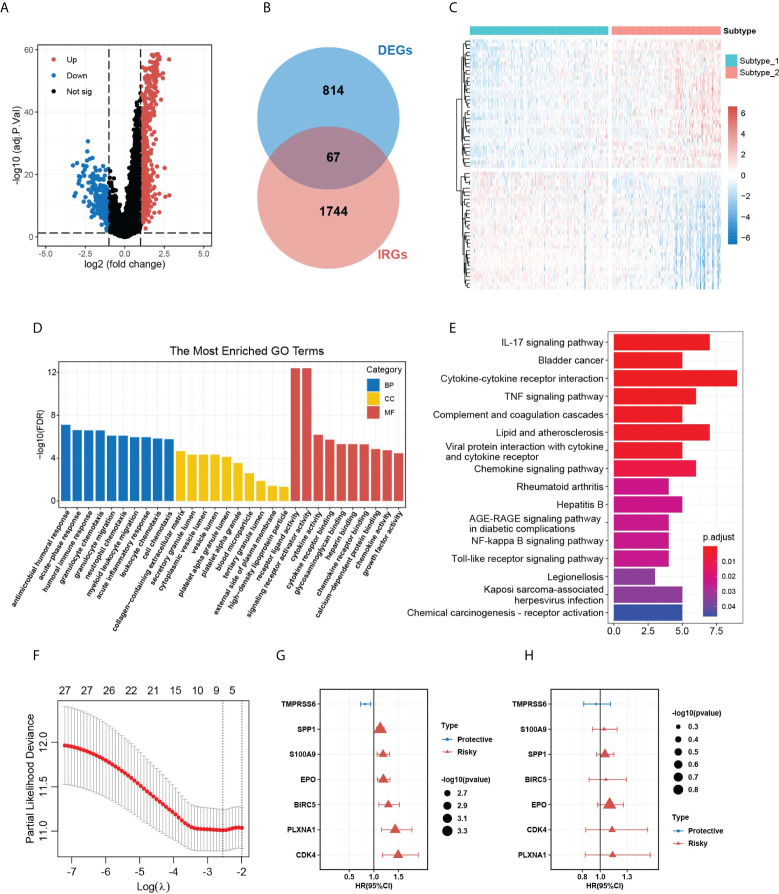
DEG analysis in the two HCC subtypes and the construction of HGSIS. **(A)** Volcano plot of 881 DEGs between the two HCC subtypes. **(B)** Venn plot showing 67 immune-related DEGs. **(C)** Heatmap of the IRDEGs in the two subtypes. **(D, E)** GO and KEGG enrichment analysis for the 67 IRDEGs. **(F)** Selection of the tuning parameter (lambda) in the LASSO model by 10-fold cross-validation. **(G, H)** Hazard ratio with 95% CI of the seven genes in the HGSIS signature computed by UniCox and MultiCox, respectively. HCC, hepatocellular carcinoma. GO, gene oncology; KEGG, Kyoto Encyclopedia of Genes and Genomes. HGSIS, hallmark-guided subtypes-based immunologic signature. IRDEGs, immune-related DEGs.

The significant difference in OS outcomes between the two subtypes prompted us to pick out the DEGs between them. Using the package “limma”, we detected 881 DEGs between subtype 1 and subtype 2, which intersected with 1,811 immune-related genes from the ImmPort database, and 67 overlapping immune-related DEGs (IRDEGs) were identified (**Figures 3A, B** and **Supplementary Table 2**). The expression heatmap of IRDEGs in the two subtypes is shown in **Figure 3C**. GO enrichment analysis revealed that the most significant terms enriched by these IRDEGs were the biological process (BP) of antimicrobial humoral response, cellular component (CC) of collagen-containing extracellular matrix, and molecular function (MF) of receptor-ligand activity and signaling receptor activator activity (**Figure 3D**). For the KEGG analysis, IRDEGs mostly participated in the pathway of cytokine–cytokine receptor interaction (**Figure 3E**). Subsequently, we inputted the IRDEGs into UniCox regression analysis and found 28 significant prognostic IRDEGs with a *p*-value lower than 0.05 (**Supplementary Table 3**). Next, we conducted LASSO Cox regression with the 28 genes and acquired seven robust genes (TMPRSS6, SPP1, S100A9, EPO, BIRC5, PLXNA1, and CDK4) that were significantly correlated with the OS of HCC patients, and the selection of the tuning parameter in the LASSO model is shown in **Figure 3F**. The seven genes were subsequently incorporated into an HGSIS model for predicting the prognosis of HCC. **Figures 3G, H** showed the UniCox and MultiCox results of the selected seven genes with the corresponding hazard ratio (HR) and statistical significance.

### Evaluation and validation of HGSIS

**Figure 4:**
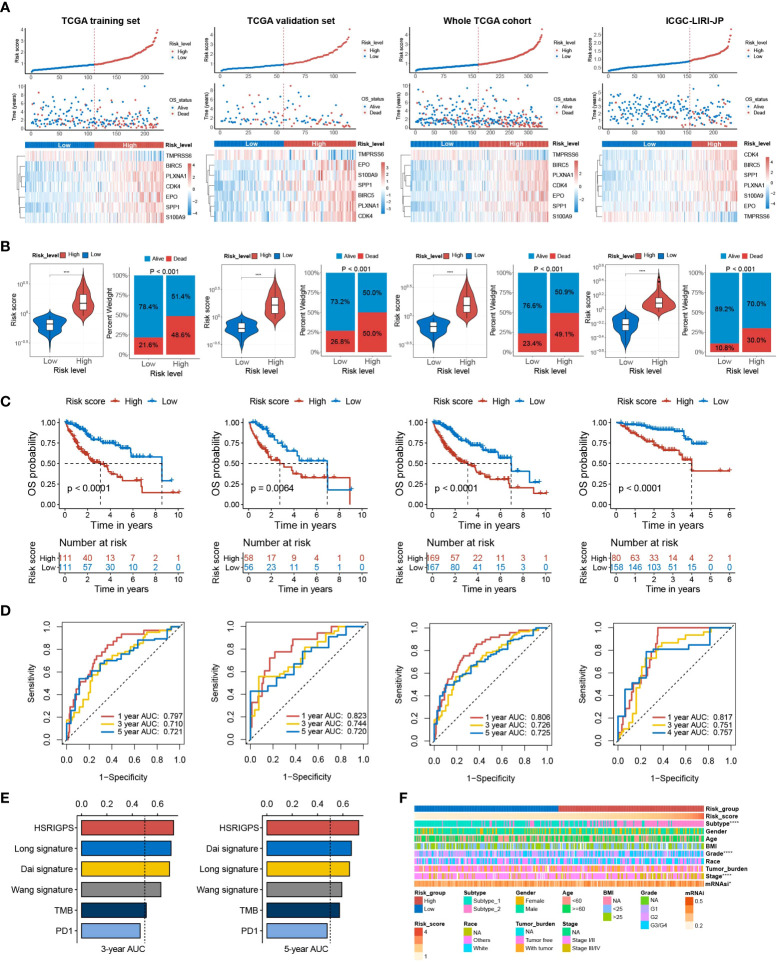
Prognostic value of HGSIS for OS in HCC patients. **(A)** Risk score distribution, survival status, and the expression of seven HGSIS signature genes for patients in the low- and high-risk groups from four datasets (TCGA training set, TCGA validation set, whole TCGA cohort, and ICGC-LIRI-JP cohort). **(B)** Risk score and mortality rates of patients in the low- and high-risk groups from four datasets. **(C)** Kaplan–Meier survival plots of the low- and high-risk groups from four datasets for OS. **(D)** tROC curves of HGSIS in the four datasets. **(E)** The comparison of AUC values for the 3-year and 5-year survival between HGSIS and other published signatures or common immunotherapeutic biomarkers. **(F)** Correlation analysis between HGSIS and multiple clinicopathological traits. **** means *p* < 0.0001.

Based on the median risk score of HGSIS, HCC patients from different datasets were classified as high- or low-risk groups (**Figure 4A**). According to the corresponding prognostic data, the high-risk groups of the TCGA training set, TCGA validation set, whole TCGA cohort, and ICGC-LIRI-JP cohort all had higher mortality (**Figure 4B**). Kaplan–Meier analysis showed that high-risk patients had exceedingly lower OS rates relative to low-risk patients in different datasets (**Figure 4C**). Additionally, the time-dependent receiver operating characteristic (tROC) curve analysis was applied to evaluate the accuracy of the HGSIS model. As shown in **Figure 4D**, the area under the ROC curve (AUC) was 0.797, 0.710, and 0.721 in 1-year, 3-year, and 5-year survival, respectively, for the TCGA training set. Interestingly, the AUC values for all the three validation datasets were even higher than the training set, suggesting that HGSIS had excellent performance in predicting the OS of HCC. In comparison with other published immune-related signatures and widely used biomarkers of cancer immunotherapy, HGSIS achieved higher predictive accuracy (**Figure 4E**). Moreover, to explore the potential relationship between HGSIS and multiple clinicopathological traits, correlation analysis was conducted and it revealed that HCC subtype, tumor grade, stage, and mRNAsi were significantly correlated with HGSIS (**Figure 4F** and **Supplementary Figure 3**). Stratification analysis for clinicopathological traits demonstrated the extra predictive value of HGSIS (**Supplementary Figure 4**). Additionally, we examined the potential of HGSIS in predicting the DFS, PFS, and DSS of HCC patients, and it revealed similar results to that of OS analysis (**Supplementary Figure 5**). Taken together, all these data presented above convincingly indicated the strong prognostic-prediction capability of HGSIS.

### Establishment of the HGSIS-integrated nomogram

**Figure 5:**
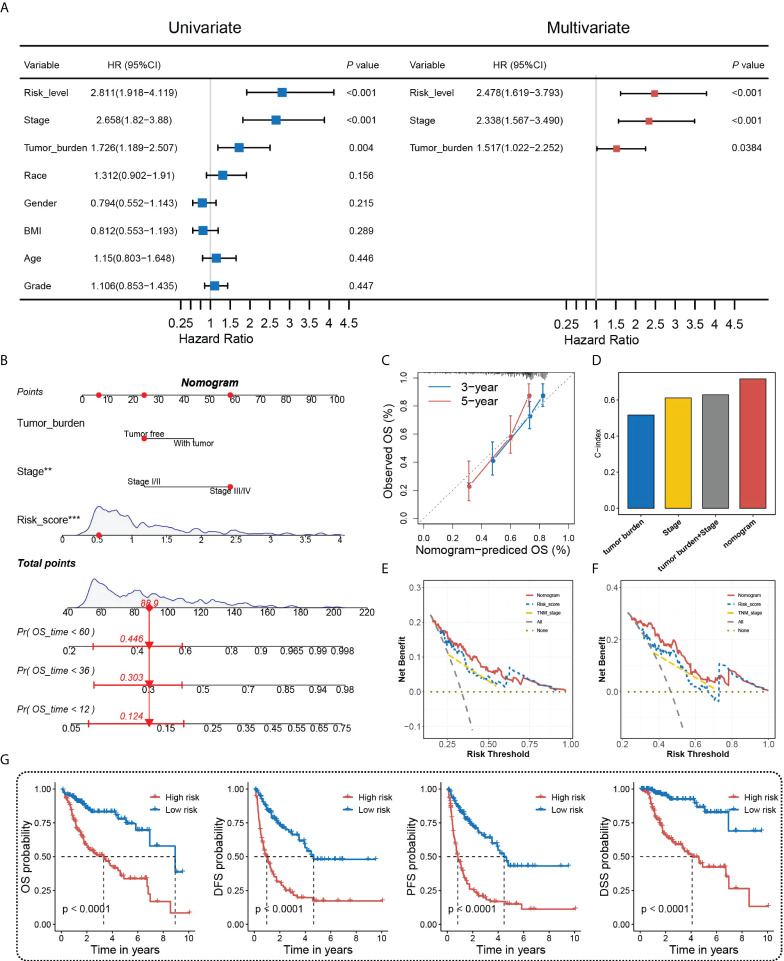
Nomogram construction and assessment. **(A)** Univariate and multivariate Cox regression analyses of HGSIS and other clinicopathological traits for OS in HCC patients. **(B)** Nomogram built by HGSIS, stage, and tumor burden to predict 1-,3- and 5-year OS in HCC patients. **(C)** Calibration plot of the nomogram. **(D)** C-index values of the nomogram and clinicopathological traits. **(E, F)** Comparison of net benefits of each model for 3-year **(E)** and 5-year **(F)** OS. **(G)** Kaplan–Meier survival analysis of the integrated nomogram for OS, DFS, PFS, and DSS in HCC patients.

Accurate and individualized prediction of the postoperative mortality risk of HCC patients has been a tough challenge in clinical decision-making. In this case, we considered constructing a novel nomogram combining HGSIS and multiple clinicopathological traits to provide an accurate and quantitative prognosis-predictive tool for HCC patients. Univariate and multivariate Cox analyses on HCC prognosis with HGSIS and clinicopathological factors were at first carried out using the whole TCGA cohort. As shown in **Figure 5A**, the HGSIS risk model, stage, and tumor burden were significantly high-risk factors for HCC in both univariate and multivariate Cox analysis, indicating that HGSIS was an independent prognostic indicator [HR (95% CI) = 2.478 (1.619−3.793), *p* < 0.001]. By integrating the three parameters, we constructed a prognostic nomogram to predict the 1-, 3-, and 5-year survival in HCC patients (**Figure 5B**). Calibration curves of the nomogram for the predicted and observed 3- and 5-year OS are shown in **Figure 5C**, suggesting the good consistency of the nomogram. Meanwhile, comparing with stage, tumor burden, and the combination of both, the HGSIS-integrated nomogram had the highest C-index, representing its best predictive accuracy (**Figure 5D**). In 3- and 5-year OS prediction for HCC patients, the nomogram showed the highest net benefit over most of the risk thresholds (**Figures 5E, F**). Furthermore, we divided HCC patients into high- and low-risk groups based on the median score of the HGSIS-integrated nomogram, and remarkably elevated OS, DFS, PFS, and DSS rates were observed in the low-risk group (**Figure 5G**). All these findings indicate that the HGSIS-integrated nomogram can serve as a powerful and valuable tool for individualized OS survival prediction in HCC patients.

### Genomic characteristics and regulatory mechanisms of the HGSIS-defined subgroups in HCC

**Figure 6:**
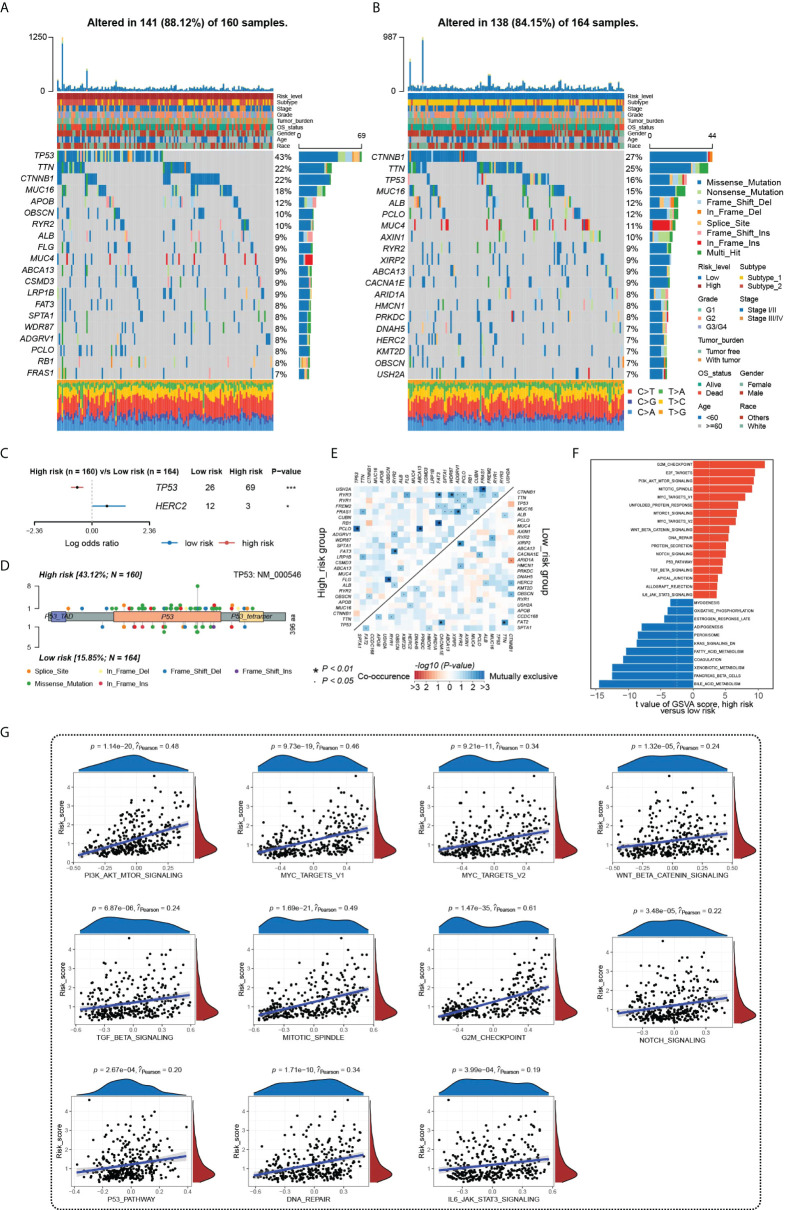
Underlying molecular mechanisms of HGSIS. **(A, B)** Oncoprint analysis of the high-risk **(A)** and low-risk groups **(B)**. **(C)** Forest plot showing genes mutated differentially in patients of the low- and high-risk groups. **(D)** Lollipop plot of mutation sites of TP53. **(E)** Interaction effect of 25 mutated genes in the low- and high-risk groups. **(F)** Distinct hallmark pathways between the two HGSIS risk groups. **(G)** Correlation analysis between 11 oncogenic hallmark pathways and HGSIS-based risk score, respectively. * means *p* < 0.05; *** means *p* < 0.001.

We further analyzed the underlying molecular mechanisms of HGSIS on the landscape of somatic mutation and hallmark pathway enrichment. We firstly examined the 20 genes with the highest mutation frequency in the low- and high-risk groups and the oncoplots showed that the most mutated genes were TP53 (43%) and CTNNB1 (27%) in the two different risk groups, respectively. Meanwhile, four genes (TP53, TTN, CTNNB1, and MUC16) simultaneously had high mutation frequencies in both two groups (**Figures 6A, B**). The summary of the mutation information is shown in **Supplementary Figure 6**. Fisher’s exact test was applied to extract the distinct mutation status between two groups and the forest plot showed that TP53 mutated more frequently while HERC2 mutation occurred less in the high-risk group significantly (**Figure 6C**). Moreover, considering that TP53 was the most notable mutated gene, a lollipop chart was established to reveal the detailed mutation sites of TP53, and more missense mutation was observed in the high-risk group (**Figure 6D**). The co-occurrences and mutual exclusions of the top 25 mutated genes in two risk groups were also shown in **Figure 6E**. To unravel the underlying transcriptomic mechanisms of HGSIS, we calculated the GSVA scores of 50 hallmark pathways in the low- and high-risk groups, respectively, to identify the key hallmark pathways associated with HGSIS, the integral landscape of which is shown in **Supplementary Figure 7**. Of the 50 hallmark pathways, 28 were found to be of significant difference between two risk groups, of which 17 hallmark pathways were upregulated while 11 hallmark pathways were downregulated in the high-risk group (**Figure 6F**). Notably, all the 11 oncogenic hallmark pathways that were upregulated in the HGSIS high-risk group were positively correlated to the HGSIS model-based risk score significantly (**Figure 6G**), indicating the tight linkage between HGSIS and those well-known oncogenic pathways.

### HGSIS was associated with the immune status in the HCC tumor microenvironment

**Figure 7:**
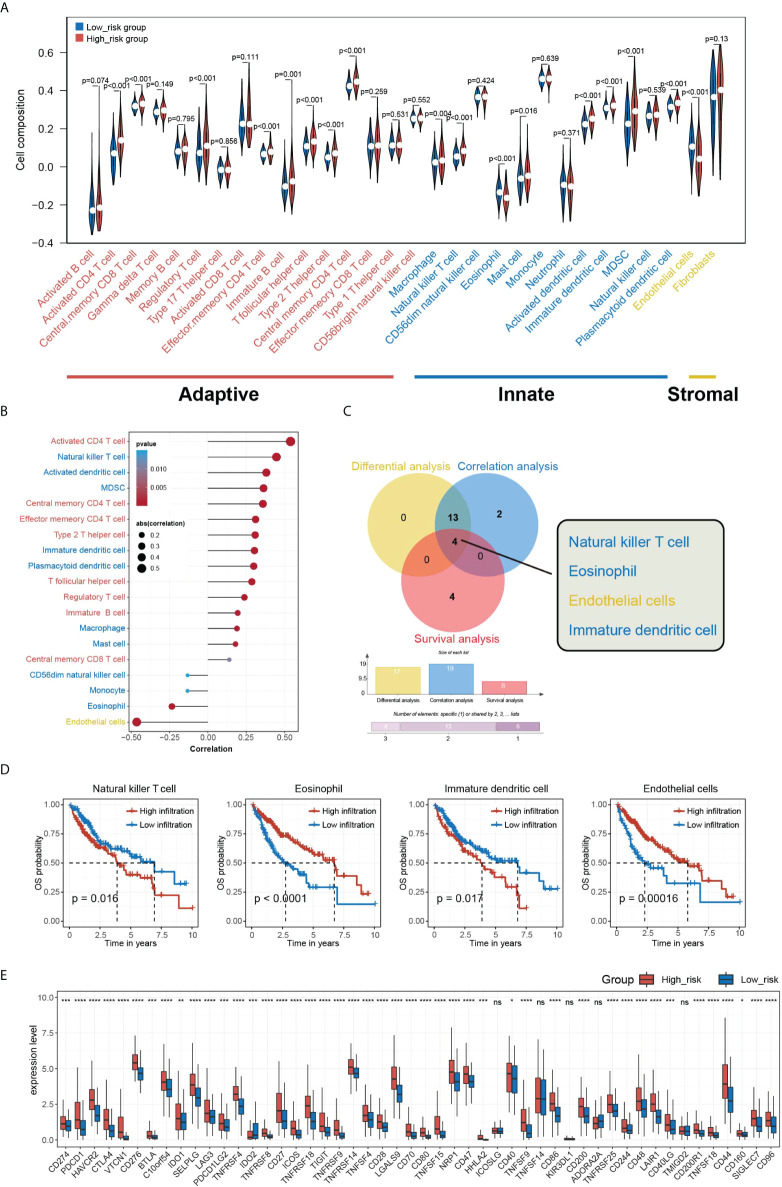
Overview of HGSIS-related immune infiltration. **(A)** Violin plot presenting the relative composition of multiple cell types in the low- and high-risk groups. **(B)** Correlation analysis of immune infiltration and HGSIS risk score. **(C)** Venn diagram revealing the four intersected cell types among differential analysis (yellow), correlation analysis (blue), and survival analysis (red). **(D)** Kaplan–Meier survival analysis of the four TME cell types. **(E)** Expression levels of common immune checkpoints in two HGSIS risk groups. TME, tumor microenvironment. * means *p* < 0.05; ** means *p* < 0.01; *** means *p* < 0.001; **** means *p* < 0.0001; ns, no significance.

To further examine the potential clinical value of HGSIS, we outlined the immune cells’ infiltration profile of the whole TCGA cohort by ssGSEA, a reliable and popular algorithm computing the relative proportion of 28 types of immune cells and two types of stromal cells in TME. As **Figure 7A** shows, the HGSIS risk group was significantly correlated with most infiltrating immune cell types including activated CD4 T cell, central memory CD8 T cell, regulatory T cell, effector memory CD4 T cell, immature B cell, T follicular helper cell, type 2 T helper cell, central memory CD4 T cell, macrophage, natural killer T cell, eosinophil, mast cell, activated dendritic cell, immature dendritic cell, MDSC, and plasmacytoid dendritic cell. Moreover, we performed correlation analysis on HGSIS risk score and TME cells, and as the result shows, 19 cell types were isolated in association with HGSIS (**Figure 7B**). Additionally, UniCox analysis found that nine types of TME cells were significantly associated with the prognosis of HCC (**Supplementary Table 4**). Combining the results of differential analysis, correlation analysis, and survival analysis of the 30 TME cell types, we plotted the Venn diagram exhibiting the four overlapping cell types (natural killer T cell, eosinophil, endothelial cells, and immature dendritic cell) (**Figure 7C**). **Figure 7D** exhibits the Kaplan–Meier curves indicating the significant implications of the four cell types for the OS of HCC patients. Meanwhile, with great anticipation, we found that the expression of the vast majority of immune checkpoints in HCC patients was significantly correlated with HGSIS, suggesting that HGSIS had the potential to predict the immune checkpoints’ expression level broadly in HCC (**Figure 7E**).

### Evaluation of HGSIS in predicting therapy response potential

**Figure 8:**
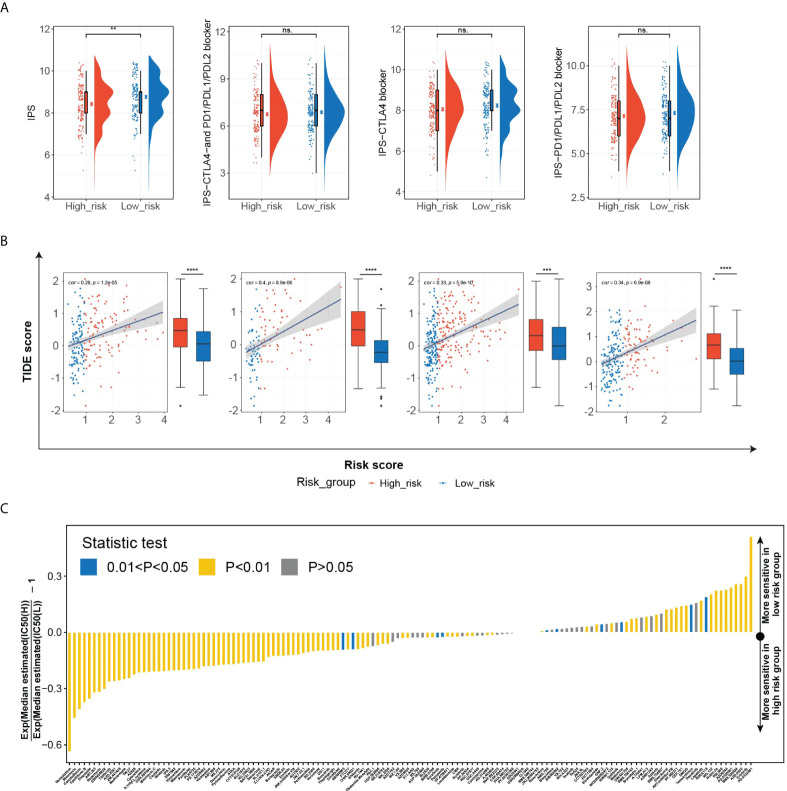
The potential role of HGSIS in predicting therapeutic sensitivity. **(A)** The correlation between HGSIS and IPS based on the whole TCGA cohort. **(B)** Distribution of TIDE scores in the TCGA training set, TCGA validation set, whole TCGA cohort, and ICGC-LIRI-JP dataset. **(C)** Estimation of 138 drugs’ normalized IC_50_ values. **p < 0.01, ***p < 0.001, ****p < 0.0001, ns, not significant.

The significant correlation between HGSIS and multiple immune checkpoints was of great interest to us, especially as immune checkpoint therapy held promise for the clinical treatment of cancer nowadays. We first explored the relationship between HGSIS and immunophenoscore (IPS) in HCC patients. IPS was well-known to be capable of predicting immune checkpoint therapy response, based on the evaluation of the pivotal immune-related gene expression. As shown in **Figure 8A**, the IPS score was significantly elevated in the low-risk group, representing higher sensitivity to immunotherapy. Although the scores of IPS-CTLA4 and PD1/PD-L1/PD-L2 blocker, IPS-CTLA4 blocker, and IPS-PD1/PD-L1/PD-L2 blocker were not statistically associated with HGSIS, the low-risk group tended to have increased scores than the high-risk group. We further measured the TIDE scores in HCC patients of the TCGA training set, the TCGA validation set, the whole TCGA cohort, and the ICGC-LIRI-JP dataset, and the low-risk groups all had significantly lower TIDE scores than the high-risk groups, suggesting that the patients in the low-risk groups were predicted to have better responses to immunotherapy (**Figure 8B**). In addition, we used the “pRRophetic” algorithm to estimate the IC_50_ values of 138 drugs for not only immunotherapy but also chemotherapy and targeted therapy for HCC patients (**Figure 8C**). Interestingly, we found that high-risk patients might be sensitive to more drugs than low-risk patients (**Figure 8C** and **Supplementary Figure 8**).

### KIF2C is a key target of HGSIS

**Figure 9:**
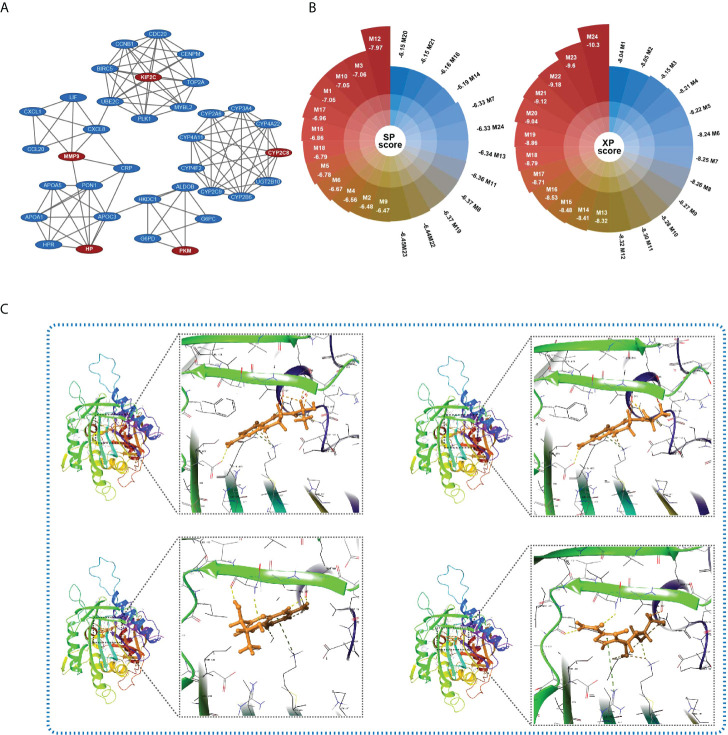
Key target identification and candidate small molecule prediction. **(A)** Five clusters identified by the MCODE app of Cytoscape 3.8.2. Red ovals represent “seed” nodes and blue ovals represent “clustered” nodes. **(B)** The docking score (XP and SP score) of the top 24 small compounds that bind to KIF2C with the lowest total energy score. **(C)** Structures and docking models of the top four small compounds (lactoyl-ph4, dihydrobiopterin, 7-biopterin, and mizoribine) and the active site of KIF2C.

To identify the key targets relating to HGSIS, we utilized the limma package to screen the DEGs between the high- and low-risk groups of the whole TCGA cohort. Consequently, 85 genes were significantly downregulated while another 85 genes were upregulated in the high-risk group (**Supplementary Table 5**). With the strict criterion of a combined score of >0.7, we constructed a PPI network of 95 nodes and 212 edges (**Supplementary Figure 9**). Furthermore, the MCODE app identified five clusters (default parameters) of the network. As shown in **Figure 9A**, KIF2C, HP, PKM, MMP9, and CYP2C8 were recognized as the “seed” nodes of these clusters (red ovals represent “seed” nodes, and blue ovals represent “clustered” nodes). Interestingly, four clusters were highly connected by driver oncogenes of HCC or immunologic genes such as CXCL8, UBE2C, and MMP9. Notably, we observed the most conspicuous cluster that consisted of several hub genes of HCC (CCNB1, CDC20, TOP2A, and UBE2C) (23, 24). Thus, the “seed” node of this cluster, KIF2C, was considered as the key target of HGSIS, and its crucial role in the discovery of putative drugs is worth looking into.

### Candidate small molecule prediction

Molecular docking is an effective computational method that provides insights into molecular interactions between candidate drugs and proteins. In the present study, we further predicted possible small molecules that may bind to the key target of HGSIS, KIF2C, *via in silico* molecular docking. As shown in **Figure 9B**, 24 small compounds were successfully filtered out from a public library that contains a large number of small molecules from the ZINC 15 database, which includes 10 commercially available molecules that were considered as potential affinity ligands of KIF2C protein (**Supplementary Table 6**). The 3D interaction diagrams of the 10 docking models showing the detailed binding energy are displayed in **Figure 9C** and **Supplementary Figure 10**. The interaction diagram of lactoyl-ph4 at the binding pocket of KIF2C suggested the formation of hydrogen bonds with key residues GLN-475, VAL-547, and ASP-550. Similarly, dihydrobiopterin also relied on hydrogen bonds between the active site and several amino acid residues to remain its high affinity with KIF2C.

## Discussions

Like most malignancies (54–57), the classification of distinct subtypes of HCC has been widely recognized in clinical practice (58–60). To date, with the increasing knowledge of the HCC TME, the exploration of the intra-tumoral immune ecosystem has been extensively involved in the study of HCC heterogeneity (61–63). Therefore, the new classification of biologically meaningful HCC subtypes gained widespread interest from either researchers or pathologists, contributing to the development of clinically useful biomarkers or signatures to predict the prognosis of HCC more precisely and individually.

In this study, we firstly employed GSVA to compute the GSVA scores of 50 hallmark gene sets that represent predefined specific gene signatures with biological significance. Robust prognostic hallmark gene sets were comprehensively identified and used to pick out distinct HCC subtypes *via* consensus clustering. Based on the hallmark-guided subtypes, we successfully developed and validated a prognostic gene signature of HCC, i.e., HGSIS. Multivariate analysis confirmed that HGSIS was an independent factor for the prediction of HCC patients’ OS survival, and the established nomogram showed increased accuracy and great potential in clinical practice.

As an advanced computational method, GSVA is one of the best and most up-to-date algorithms throwing light on the discovery of subtle pathway activity changes in a given population by condensing gene expression profiles into pathways. Herein, we defined two distinct HCC subtypes with 15 prognostic hallmark gene sets, most of which were metabolism-related and immune-related. For example, glycolysis gene sets included genes encoding proteins involved in glycolysis and gluconeogenesis, defined as biological processes responsible for the regulation of proliferation, immune evasion, invasion, metastasis, angiogenesis, and drug resistance in HCC (64). The PI3K/AKT/mTOR pathway was linked to drug resistance and the occurrence of HCC in a recent clinical study (65). Based on the hallmark-guided subtypes of HCC, we focused on the immune-related genes expressed differently between two subtypes, and 67 IRDEGs were extracted from 881 DEGs. HGSIS construction was carried out with UniCox and LASSO Cox analysis on 67 IRDEGs, leading to a seven-gene prognostic signature (TMPRSS6, SPP1, S100A9, EPO, BIRC5, PLXNA1, and CDK4). TMPRSS6 was reported to express much lower in HCC cell lines when compared to normal liver samples, which was consistent with our HGSIS model, presenting a protective role of TMPRSS6 in HCC (66). Ma et al. found that SPP1 expression was tightly linked to the TME reprogramming and tumor progression in response to therapy by single-cell transcriptomic analysis (67). S100A9, PLXNA1, and EPO were also reported as candidates for HCC prognostic signatures, implying their subtle effects on the disease progression of HCC patients (68–70). Moreover, BIRC5 was also found to be highly expressed in liver cancer (71). CDK4 was a well-recognized oncogene, and it was encouraging that Palbociclib, a CDK4 inhibitor, showed significant benefit in preclinical models of HCC (72). In summary, HGSIS was a model that fitted well with HCC. After being validated internally and externally, HGSIS showed outstanding prediction accuracy for HCC patients, and the HGSIS high-risk group obviously presented a worse prognosis.

A growing number of prognostic gene classifiers have been developed to evaluate the mortality risks of HCC patients, most of which were based on limited gene sets or DEGs between tumor and adjacent normal samples. For instance, Liu et al. discovered a six-gene signature presenting a strong ability for differentiating HCC tumors and normal tissues (73). Li et al. constructed an lncRNA signature for estimating OS of HCC patients (74). However, few previous studies combined multi-gene sets to recognize HCC subtypes with distinct survival outcomes, and here, we, for the first time, adopted the hallmark-guided subtype-based identification of immunologic gene signatures to predict the OS of HCC patients. Importantly, the comparison of AUC values between HGSIS and other reported signatures or immunotherapeutic targets demonstrated the reliability of our novel strategy in building a prognostic classifier. Thus, our study provided a novel perspective for the first time, that hallmark-gene set-based cancer subtypes could make a firm basis for clinical classifiers construction. Another strength of the present study was the integrative and combined strategy that included GSVA, unsupervised clustering, UniCox, and LASSO-Cox, which was more effective and reliable than that where only one or two algorithms were applied. Thirdly, unlike most studies that did not assess the feasibility of prognostic signatures for drug prediction, we employed PPI construction, key genes identification, and molecular docking to select the most probable small compounds from a large number of potential drugs, some of which had been reported to have an anti-cancer effect in several cancers. Furthermore, it cannot be denied that, at all levels from molecular alterations to histopathologic diversity, more comprehensive research would provide better references for understanding the mechanisms of drug resistance or treatment inefficiencies in HCC patients (75).

Tumor mutational burden (TMB) has been confirmed to be related to the immunotherapy effectiveness and prognosis in various malignancies; however, its underlying mechanism in HCC remained unclear (76). Therefore, we used maftools to evaluate the mutation status in HGSIS low- and high-risk groups. TP53 showed the highest mutation frequency (43%) in the high-risk group while CTNNB1 mutated most frequently in the low-risk group. Consistent with previous reports, TP53 and CTNNB1 are the most common genetic alterations in HCC (77, 78). Interestingly, by exome sequencing analysis, a remarkable study found that alcohol-related HCC was significantly associated with CTNNB1 mutation, and TP53 mutation frequently occurred in HBV-related HCC (79). Logically, more alcoholics might be classified into the low-risk group and more patients infected with HBV might be related to the high-risk group. Several well-recognized oncogenic signaling pathways were transcriptionally activated in the high-risk group including the most researched TP53 pathway, MYC pathway, Wnt/β-Catenin pathway, PI3K/AKT/mTOR pathway, and Notch pathway (64, 80–82). Thus, HGSIS was capable of separating groups of different mutation statuses in HCC.

Given that tumor-infiltrating immune cells constituting the major component of TME are associated with the prognosis and immunotherapy efficacy of HCC (83), ssGSEA algorithm was used to estimate the relative proportion of TME cell types in the HGSIS low- and high-risk groups. The relative abundance of 17 TME cell types differed significantly between the two subgroups. A large body of studies has illustrated that regulatory T cells (Tregs) played an immunosuppressive role in TME and were associated with a poor prognosis of HCC (84–86). Unsurprisingly, our study found that more Tregs infiltrated in TME of patients from the high-risk group. In the past decade, immunotherapy, especially ICIs, has become a highlight research direction for the treatment of broad-spectrum malignancies. Moreover, as HCC usually arises in the context of virus-related chronic inflammation, immunotherapy is likely to be an ideal therapeutic option for HCC. In our study, differential analysis revealed relatively higher levels of immune checkpoint expression in the HGSIS high-risk group, indicating that patients in the high-risk group were accompanied by a worse anti-TIME. For the results of IPS and TIDE analysis, we found that the low-risk group had higher IPS scores while having a lower TIDE score, and higher IPS and lower TIDE corresponded to a better prognosis. On the other hand, we estimated the sensitivity of 138 drugs included in immunotherapy, chemotherapy, and targeted therapy and found that the high-risk group was sensitive to more drugs. Taken together, the HGSIS model could potentially serve as a reference for individualized immunotherapy design.

To screen small molecules used as potential drugs to reverse the high risk of HCC patients, we analyzed the DEGs between the two HGSIS risk groups, which were utilized to build an interactive PPI network. From the network, KIF2C was identified to be the key node that connected to several hub oncogenes of HCC. Notably, KIF2C has been revealed as a prognostic biomarker in endometrial cancer and correlated with the infiltration level of CD8+ T cells (87). In HCC, Wei et al. disclosed that KIF2C is associated with a poor prognosis of HCC and interacts with TBC1D7 to enhance the mTORC1 signal transduction (88). In the present study, using the structure-based approach, we identified 10 purchasable small compounds that may bind well to KIF2C from a large number of small compounds. Among them, mizoribine is a novel and effective immunosuppressant that inhibits the activity of HCV RNA replication, and it is also a selective inhibitor of inosine-5’-monophosphate dehydrogenase (IMPDH) that has been clinically used throughout Asia. Importantly, mizoribine exhibits far superior antitumor activity compared with several FDA-approved IMPDH inhibitors, and mizoribine treatment shows a more durable antitumor response than the mTOR inhibitor rapamycin (89). In addition, mitoxantrone, a firmly established inhibitor of type II topoisomerase and protein kinase C (PKC), is reported to exert its anti-cancer effect in lymphomas, leukemias, and breast, colorectal, and prostate cancers (90–97). Although more preclinical investigations need to be completed to validate their anti-cancer activities, the selected small molecules hold great potential in the future application of clinical treatment of HCC.

The study’s limitations should be noted. First, real-world evidence from large clinical cohorts is required to test the utility and significance of HGSIS for further clinical practice. Second, more *in vitro* and *in vivo* experiments were needed to unveil the molecular underpinnings in terms of HGSIS, as well as the effectiveness of the putative drugs for treating HCC.

## Conclusion

In conclusion, two hallmark-guided subtypes of HCC were extensively identified. Based on the HCC subtypes, an immunological prognostic signature, HGSIS, was developed and validated, which was associated with tumor immune phenotypes, distinct genomic landscapes, and therapeutic responses. Combining HGSIS, PPI network construction, and structure-based *in silico* docking, we also predicted candidate small drugs that may bind to the key target of HGSIS, which act as potential drugs for HCC. Therefore, our study provides a novel perspective to recognizing cancer subtypes with clinical implications, which serves as an entry point for the construction of better risk classifiers to design personalized treatment to prolong patients’ survival.

## Data availability statement

Publicly available datasets were analyzed in this study. This data can be found here: https://portal.gdc.cancer.gov/and https://icgc.org/.

## Author contributions

YZ, and GL conceived the study. YZ, CG, and YT contributed to data collection, analysis, and interpretation. YZ and CG completed the drafting of the manuscript. ZY contributed to the revision of the manuscript. All authors contributed to the article and approved the submitted version.

## Acknowledgments

We acknowledge The Cancer Genome Atlas (TCGA) and International Cancer Genome Consortium (ICGC) for data sharing.

## Conflict of interest

The authors declare that the research was conducted in the absence of any commercial or financial relationships that could be construed as a potential conflict of interest.

## Publisher’s note

All claims expressed in this article are solely those of the authors and do not necessarily represent those of their affiliated organizations, or those of the publisher, the editors and the reviewers. Any product that may be evaluated in this article, or claim that may be made by its manufacturer, is not guaranteed or endorsed by the publisher.

## References

[1] Gong J Li R Chen Y Zhuo Z Chen S Cao J. Hcc subtypes based on the activity changes of immunologic and hallmark gene sets in tumor and nontumor tissues. Brief Bioinform (2021) 22(5):bbaa427. doi: 10.1093/bib/bbaa427 33515024

[2] Llovet JM Kelley RK Villanueva A Singal AG Pikarsky E Roayaie S. Hepatocellular carcinoma. Nat Rev Dis Primers (2021) 7(1):6. doi: 10.1038/s41572-020-00240-3 33479224 PMC13537433

[3] El Jabbour T Lagana SM Lee H. Update on hepatocellular carcinoma: Pathologists' review. World J Gastroenterol (2019) 25(14):1653–65. doi: 10.3748/wjg.v25.i14.1653 PMC6465943 31011252

[4] Zhang Q Lou Y Yang J Wang J Feng J Zhao Y. Integrated multiomic analysis reveals comprehensive tumour heterogeneity and novel immunophenotypic classification in hepatocellular carcinomas. Gut (2019) 68(11):2019–31. doi: 10.1136/gutjnl-2019-318912 PMC6839802 31227589

[5] Ho DW Tsui YM Sze KM Chan LK Cheung TT Lee E. Single-cell transcriptomics reveals the landscape of intra-tumoral heterogeneity and stemness-related subpopulations in liver cancer. Cancer Lett (2019) 459:176–85. doi: 10.1016/j.canlet.2019.06.002 31195060

[6] Li L Wang H. Heterogeneity of liver cancer and personalized therapy. Cancer Lett (2016) 379(2):191–7. doi: 10.1016/j.canlet.2015.07.018 26213370

[7] Farmer DG Rosove MH Shaked A Busuttil RW. Current treatment modalities for hepatocellular carcinoma. Ann Surg (1994) 219(3):236–47. doi: 10.1097/00000658-199403000-00003 PMC1243131 8147605

[8] Liang JY Wang DS Lin HC Chen XX Yang H Zheng Y. A novel ferroptosis-related gene signature for overall survival prediction in patients with hepatocellular carcinoma. Int J Biol Sci (2020) 16(13):2430–41. doi: 10.7150/ijbs.45050 PMC7378635 32760210

[9] Zhang B Tang B Gao J Li J Kong L Qin L. A hypoxia-related signature for clinically predicting diagnosis, prognosis and immune microenvironment of hepatocellular carcinoma patients. J Transl Med (2020) 18(1):342. doi: 10.1186/s12967-020-02492-9 32887635 PMC7487492

[10] Hu B Yang XB Sang XT. Construction of a lipid metabolism-related and immune-associated prognostic signature for hepatocellular carcinoma. Cancer Med (2020) 9(20):7646–62. doi: 10.1002/cam4.3353 PMC7571839 32813933

[11] Kurebayashi Y Ojima H Tsujikawa H Kubota N Maehara J Abe Y. Landscape of immune microenvironment in hepatocellular carcinoma and its additional impact on histological and molecular classification. Hepatology (2018) 68(3):1025–41. doi: 10.1002/hep.29904 29603348

[12] Hanahan D Coussens LM. Accessories to the crime: Functions of cells recruited to the tumor microenvironment. Cancer Cell (2012) 21(3):309–22. doi: 10.1016/j.ccr.2012.02.022 22439926

[13] Sangro B Sarobe P Hervas-Stubbs S Melero I. Advances in immunotherapy for hepatocellular carcinoma. Nat Rev Gastroenterol Hepatol (2021) 18(8):525–43. doi: 10.1038/s41575-021-00438-0 PMC8042636 33850328

[14] Sangro B Gomez-Martin C de la Mata M Inarrairaegui M Garralda E Barrera P. A clinical trial of ctla-4 blockade with tremelimumab in patients with hepatocellular carcinoma and chronic hepatitis c. J Hepatol (2013) 59(1):81–8. doi: 10.1016/j.jhep.2013.02.022 23466307

[15] Finn RS Qin S Ikeda M Galle PR Ducreux M Kim T-Y. Imbrave150: Updated overall survival (Os) data from a global, randomized, open-label phase iii study of atezolizumab (Atezo) plus bevacizumab (Bev) versus sorafenib (Sor) in patients (Pts) with unresectable hepatocellular carcinoma (Hcc). J Clin Oncol (2021) 39(3):267. doi: 10.1200/JCO.2021.39.3_suppl.267

[16] Palucka AK Coussens LM. The basis of oncoimmunology. Cell (2016) 164(6):1233–47. doi: 10.1016/j.cell.2016.01.049 PMC4788788 26967289

[17] Hanzelmann S Castelo R Guinney J. Gsva: Gene set variation analysis for microarray and rna-seq data. BMC Bioinf (2013) 14:7. doi: 10.1186/1471-2105-14-7 PMC3618321 23323831

[18] Subramanian A Tamayo P Mootha VK Mukherjee S Ebert BL Gillette MA. Gene set enrichment analysis: A knowledge-based approach for interpreting genome-wide expression profiles. Proc Natl Acad Sci USA (2005) 102(43):15545–50. doi: 10.1073/pnas.0506580102 PMC1239896 16199517

[19] Liberzon A Birger C Thorvaldsdottir H Ghandi M Mesirov JP Tamayo P. The molecular signatures database (Msigdb) hallmark gene set collection. Cell Syst (2015) 1(6):417–25. doi: 10.1016/j.cels.2015.12.004 PMC4707969 26771021

[20] Tang Y Guo C Yang Z Wang Y Zhang Y Wang D. Identification of a tumor immunological phenotype-related gene signature for predicting prognosis, immunotherapy efficacy, and drug candidates in hepatocellular carcinoma. Front Immunol (2022) 13:862527. doi: 10.3389/fimmu.2022.862527 35493471 PMC9039265

[21] Cai WY Dong ZN Fu XT Lin LY Wang L Ye GD. Identification of a tumor microenvironment-relevant gene set-based prognostic signature and related therapy targets in gastric cancer. Theranostics (2020) 10(19):8633–47. doi: 10.7150/thno.47938 PMC7392024 32754268

[22] Malta TM Sokolov A Gentles AJ Burzykowski T Poisson L Weinstein JN. Machine learning identifies stemness features associated with oncogenic dedifferentiation. Cell (2018) 173(2):338–54 e15. doi: 10.1016/j.cell.2018.03.034 29625051 PMC5902191

[23] Zhang Y Tang Y Guo C Li G. Integrative analysis identifies key mrna biomarkers for diagnosis, prognosis, and therapeutic targets of hcv-associated hepatocellular carcinoma. Aging (Albany NY) (2021) 13:12865–95. doi: 10.18632/aging.202957 PMC8148482 33946043

[24] Tang Y Zhang Y Hu X. Identification of potential hub genes related to diagnosis and prognosis of hepatitis b virus-related hepatocellular carcinoma *Via* integrated bioinformatics analysis. BioMed Res Int (2020) 2020:4251761. doi: 10.1155/2020/4251761 33376723 PMC7744201

[25] Mayakonda A Lin DC Assenov Y Plass C Koeffler HP. Maftools: Efficient and comprehensive analysis of somatic variants in cancer. Genome Res (2018) 28(11):1747–56. doi: 10.1101/gr.239244.118 PMC6211645 30341162

[26] Bhattacharya S Andorf S Gomes L Dunn P Schaefer H Pontius J. Immport: Disseminating data to the public for the future of immunology. Immunol Res (2014) 58(2-3):234–9. doi: 10.1007/s12026-014-8516-1 24791905

[27] Wilkerson MD Hayes DN. Consensusclusterplus: A class discovery tool with confidence assessments and item tracking. Bioinformatics (2010) 26(12):1572–3. doi: 10.1093/bioinformatics/btq170 PMC2881355 20427518

[28] Chen D Huang H Zang L Gao W Zhu H Yu X. Development and verification of the hypoxia- and immune-associated prognostic signature for pancreatic ductal adenocarcinoma. Front Immunol (2021) 12:728062. doi: 10.3389/fimmu.2021.728062 34691034 PMC8526937

[29] Wang Z Wang Y Yang T Xing H Wang Y Gao L. Machine learning revealed stemness features and a novel stemness-based classification with appealing implications in discriminating the prognosis, immunotherapy and temozolomide responses of 906 glioblastoma patients. Brief Bioinform (2021) 22(5):bbab032. doi: 10.1093/bib/bbab032 33839757 PMC8425448

[30] Guo C Tang Y Zhang Y Li G. Mining tcga data for key biomarkers related to immune microenvironment in endometrial cancer by immune score and weighted correlation network analysis. Front Mol Biosci (2021) 8:645388. doi: 10.3389/fmolb.2021.645388 33869285 PMC8048410

[31] Ritchie ME Phipson B Wu D Hu Y Law CW Shi W. Limma powers differential expression analyses for rna-sequencing and microarray studies. Nucleic Acids Res (2015) 43(7):e47. doi: 10.1093/nar/gkv007 25605792 PMC4402510

[32] Yu G Wang LG Han Y He QY. Clusterprofiler: An r package for comparing biological themes among gene clusters. OMICS (2012) 16(5):284–7. doi: 10.1089/omi.2011.0118 PMC3339379 22455463

[33] Friedman J Hastie T Tibshirani R. Regularization paths for generalized linear models *Via* coordinate descent. J Stat Softw (2010) 33(1):1–22. doi: 10.18637/jss.v033.i01 20808728 PMC2929880

[34] Long J Wang A Bai Y Lin J Yang X Wang D. Development and validation of a Tp53-associated immune prognostic model for hepatocellular carcinoma. EBioMedicine (2019) 42:363–74. doi: 10.1016/j.ebiom.2019.03.022 PMC6491941 30885723

[35] Wang Z Zhu J Liu Y Liu C Wang W Chen F. Development and validation of a novel immune-related prognostic model in hepatocellular carcinoma. J Transl Med (2020) 18(1):67. doi: 10.1186/s12967-020-02255-6 32046766 PMC7011553

[36] Dai Y Qiang W Lin K Gui Y Lan X Wang D. An immune-related gene signature for predicting survival and immunotherapy efficacy in hepatocellular carcinoma. Cancer Immunol Immunother (2021) 70(4):967–79. doi: 10.1007/s00262-020-02743-0 PMC10992402 33089373

[37] Sayaman RW Saad M Thorsson V Hu D Hendrickx W Roelands J. Germline genetic contribution to the immune landscape of cancer. Immunity (2021) 54(2):367–86 e8. doi: 10.1016/j.immuni.2021.01.011 33567262 PMC8414660

[38] Thorsson V Gibbs DL Brown SD Wolf D Bortone DS Ou Yang TH. The immune landscape of cancer. Immunity (2018) 48(4):812–30 e14. doi: 10.1016/j.immuni.2018.03.023 29628290 PMC5982584

[39] Rooney MS Shukla SA Wu CJ Getz G Hacohen N. Molecular and genetic properties of tumors associated with local immune cytolytic activity. Cell (2015) 160(1-2):48–61. doi: 10.1016/j.cell.2014.12.033 25594174 PMC4856474

[40] Chalmers ZR Connelly CF Fabrizio D Gay L Ali SM Ennis R. Analysis of 100,000 human cancer genomes reveals the landscape of tumor mutational burden. Genome Med (2017) 9(1):34. doi: 10.1186/s13073-017-0424-2 28420421 PMC5395719

[41] Sun J Shi R Zhang X Fang D Rauch J Lu S. Characterization of immune landscape in papillary thyroid cancer reveals distinct tumor immunogenicity and implications for immunotherapy. Oncoimmunology (2021) 10(1):e1964189. doi: 10.1080/2162402X.2021.1964189 34513318 PMC8425706

[42] Lambrechts D Wauters E Boeckx B Aibar S Nittner D Burton O. Phenotype molding of stromal cells in the lung tumor microenvironment. Nat Med (2018) 24(8):1277–89. doi: 10.1038/s41591-018-0096-5 29988129

[43] Wang S Zhang Q Yu C Cao Y Zuo Y Yang L. Immune cell infiltration-based signature for prognosis and immunogenomic analysis in breast cancer. Brief Bioinform (2020) 22(2):2020–31. doi: 10.1093/bib/bbaa026 32141494

[44] Charoentong P Finotello F Angelova M Mayer C Efremova M Rieder D. Pan-cancer immunogenomic analyses reveal genotype-immunophenotype relationships and predictors of response to checkpoint blockade. Cell Rep (2017) 18(1):248–62. doi: 10.1016/j.celrep.2016.12.019 28052254

[45] Becht E Giraldo NA Lacroix L Buttard B Elarouci N Petitprez F. Estimating the population abundance of tissue-infiltrating immune and stromal cell populations using gene expression. Genome Biol (2016) 17(1):218. doi: 10.1186/s13059-016-1070-5 27765066 PMC5073889

[46] Wu XN Su D Mei YD Xu MQ Zhang H Wang ZY. Identified lung adenocarcinoma metabolic phenotypes and their association with tumor immune microenvironment. Cancer Immunol Immunother (2021) 70(10):2835–50. doi: 10.1007/s00262-021-02896-6 PMC10992324 33659999

[47] Wu J Li L Zhang H Zhao Y Zhang H Wu S. A risk model developed based on tumor microenvironment predicts overall survival and associates with tumor immunity of patients with lung adenocarcinoma. Oncogene (2021) 40(26):4413–24. doi: 10.1038/s41388-021-01853-y 34108619

[48] Liu Z Lu T Wang L Liu L Li L Han X. Comprehensive molecular analyses of a novel mutational signature classification system with regard to prognosis, genomic alterations, and immune landscape in glioma. Front Mol Biosci (2021) 8:682084. doi: 10.3389/fmolb.2021.682084 34307451 PMC8293748

[49] Jiang P Gu S Pan D Fu J Sahu A Hu X. Signatures of T cell dysfunction and exclusion predict cancer immunotherapy response. Nat Med (2018) 24(10):1550–8. doi: 10.1038/s41591-018-0136-1 PMC6487502 30127393

[50] Geeleher P Cox N Huang RS. Prrophetic: An r package for prediction of clinical chemotherapeutic response from tumor gene expression levels. PloS One (2014) 9(9):e107468. doi: 10.1371/journal.pone.0107468 25229481 PMC4167990

[51] Shannon P Markiel A Ozier O Baliga NS Wang JT Ramage D. Cytoscape: A software environment for integrated models of biomolecular interaction networks. Genome Res (2003) 13(11):2498–504. doi: 10.1101/gr.1239303 PMC403769 14597658

[52] Bader GD Hogue CW. An automated method for finding molecular complexes in Large protein interaction networks. BMC Bioinf (2003) 4:2. doi: 10.1186/1471-2105-4-2 PMC149346 12525261

[53] Jimenez J Doerr S Martinez-Rosell G Rose AS De Fabritiis G. Deepsite: Protein-binding site predictor using 3d-convolutional neural networks. Bioinformatics (2017) 33(19):3036–42. doi: 10.1093/bioinformatics/btx350 28575181

[54] Zhang YQ Wang WY Xue JX Xu Y Fan P Caughey BA. Microrna expression profile on solid subtype of invasive lung adenocarcinoma reveals a panel of four mirnas to be associated with poor prognosis in Chinese patients. J Cancer (2016) 7(12):1610–20. doi: 10.7150/jca.14923 PMC5039382 27698898

[55] Zhu B Tse LA Wang D Koka H Zhang T Abubakar M. Immune gene expression profiling reveals heterogeneity in luminal breast tumors. Breast Cancer Res (2019) 21(1):147. doi: 10.1186/s13058-019-1218-9 31856876 PMC6924001

[56] Marusyk A Janiszewska M Polyak K. Intratumor heterogeneity: The Rosetta stone of therapy resistance. Cancer Cell (2020) 37(4):471–84. doi: 10.1016/j.ccell.2020.03.007 PMC7181408 32289271

[57] Bruun J Kryeziu K Eide PW Moosavi SH Eilertsen IA Langerud J. Patient-derived organoids from multiple colorectal cancer liver metastases reveal moderate intra-patient pharmacotranscriptomic heterogeneity. Clin Cancer Res (2020) 26(15):4107–19. doi: 10.1158/1078-0432.CCR-19-3637 32299813

[58] Yasui K Hashimoto E Komorizono Y Koike K Arii S Imai Y. Characteristics of patients with nonalcoholic steatohepatitis who develop hepatocellular carcinoma. Clin Gastroenterol Hepatol (2011) 9(5):428–33. doi: 10.1016/j.cgh.2011.01.023 21320639

[59] Cheuk W Chan JK. Clear cell variant of fibrolamellar carcinoma of the liver. Arch Pathol Lab Med (2001) 125(9):1235–8. doi: 10.5858/2001-125-1235-CCVOFC 11520281

[60] Lee JH Choi MS Gwak GY Lee JH Koh KC Paik SW. Clinicopathologic characteristics and long-term prognosis of scirrhous hepatocellular carcinoma. Dig Dis Sci (2012) 57(6):1698–707. doi: 10.1007/s10620-012-2075-x 22327241

[61] Zhang Q He Y Luo N Patel SJ Han Y Gao R. Landscape and dynamics of single immune cells in hepatocellular carcinoma. Cell (2019) 179(4):829–45 e20. doi: 10.1016/j.cell.2019.10.003 31675496

[62] Santhakumar C Gane EJ Liu K McCaughan GW. Current perspectives on the tumor microenvironment in hepatocellular carcinoma. Hepatol Int (2020) 14(6):947–57. doi: 10.1007/s12072-020-10104-3 33188512

[63] Ringelhan M Pfister D O'Connor T Pikarsky E Heikenwalder M. The immunology of hepatocellular carcinoma. Nat Immunol (2018) 19(3):222–32. doi: 10.1038/s41590-018-0044-z 29379119

[64] Feng J Li J Wu L Yu Q Ji J Wu J. Emerging roles and the regulation of aerobic glycolysis in hepatocellular carcinoma. J Exp Clin Cancer Res (2020) 39(1):126. doi: 10.1186/s13046-020-01629-4 32631382 PMC7336654

[65] Harding JJ Nandakumar S Armenia J Khalil DN Albano M Ly M. Prospective genotyping of hepatocellular carcinoma: Clinical implications of next-generation sequencing for matching patients to targeted and immune therapies. Clin Cancer Res (2019) 25(7):2116–26. doi: 10.1158/1078-0432.CCR-18-2293 PMC6689131 30373752

[66] Dion SP Beliveau F Morency LP Desilets A Najmanovich R Leduc R. Functional diversity of Tmprss6 isoforms and variants expressed in hepatocellular carcinoma cell lines. Sci Rep (2018) 8(1):12562. doi: 10.1038/s41598-018-30618-z 30135444 PMC6105633

[67] Ma L Wang L Khatib SA Chang CW Heinrich S Dominguez DA. Single-cell atlas of tumor cell evolution in response to therapy in hepatocellular carcinoma and intrahepatic cholangiocarcinoma. J Hepatol (2021) 75(6):1397–408. doi: 10.1016/j.jhep.2021.06.028 PMC8604764 34216724

[68] Wang Y Yang Y Zhao Z Sun H Luo D Huttad L. A new nomogram model for prognosis of hepatocellular carcinoma based on novel gene signature that regulates cross-talk between immune and tumor cells. BMC Cancer (2022) 22(1):379. doi: 10.1186/s12885-022-09465-9 35397536 PMC8994280

[69] Hu B Yang XB Sang XT. Development and verification of the hypoxia-related and immune-associated prognosis signature for hepatocellular carcinoma. J Hepatocell Carcinoma (2020) 7:315–30. doi: 10.2147/JHC.S272109 PMC7667586 33204664

[70] Hu B Yang XB Sang XT. Molecular subtypes based on immune-related genes predict the prognosis for hepatocellular carcinoma patients. Int Immunopharmacol (2021) 90:107164. doi: 10.1016/j.intimp.2020.107164 33172741

[71] Chaudhary K Poirion OB Lu L Garmire LX. Deep learning-based multi-omics integration robustly predicts survival in liver cancer. Clin Cancer Res (2018) 24(6):1248–59. doi: 10.1158/1078-0432.CCR-17-0853 PMC6050171 28982688

[72] Bollard J Miguela V Ruiz de Galarreta M Venkatesh A Bian CB Roberto MP. Palbociclib (Pd-0332991), a selective Cdk4/6 inhibitor, restricts tumour growth in preclinical models of hepatocellular carcinoma. Gut (2017) 66(7):1286–96. doi: 10.1136/gutjnl-2016-312268 PMC5512174 27849562

[73] Liu GM Zeng HD Zhang CY Xu JW. Identification of a six-gene signature predicting overall survival for hepatocellular carcinoma. Cancer Cell Int (2019) 19:138. doi: 10.1186/s12935-019-0858-2 31139015 PMC6528264

[74] Li W Chen QF Huang T Wu P Shen L Huang ZL. Identification and validation of a prognostic lncrna signature for hepatocellular carcinoma. Front Oncol (2020) 10:780. doi: 10.3389/fonc.2020.00780 32587825 PMC7298074

[75] Cancer Genome Atlas Research Network. Electronic address wbe, cancer genome atlas research n. comprehensive and integrative genomic characterization of hepatocellular carcinoma. Cell (2017) 169(7):1327–41 e23. doi: 10.1016/j.cell.2017.05.046 28622513 PMC5680778

[76] Tang B Zhu J Zhao Z Lu C Liu S Fang S. Diagnosis and prognosis models for hepatocellular carcinoma patient's management based on tumor mutation burden. J Adv Res (2021) 33:153–65. doi: 10.1016/j.jare.2021.01.018 PMC8463909 34603786

[77] Calderaro J Ziol M Paradis V Zucman-Rossi J. Molecular and histological correlations in liver cancer. J Hepatol (2019) 71(3):616–30. doi: 10.1016/j.jhep.2019.06.001 31195064

[78] Candia J Bayarsaikhan E Tandon M Budhu A Forgues M Tovuu LO. The genomic landscape of Mongolian hepatocellular carcinoma. Nat Commun (2020) 11(1):4383. doi: 10.1038/s41467-020-18186-1 32873799 PMC7462863

[79] Schulze K Imbeaud S Letouze E Alexandrov LB Calderaro J Rebouissou S. Exome sequencing of hepatocellular carcinomas identifies new mutational signatures and potential therapeutic targets. Nat Genet (2015) 47(5):505–11. doi: 10.1038/ng.3252 PMC4587544 25822088

[80] Levrero M Zucman-Rossi J. Mechanisms of hbv-induced hepatocellular carcinoma. J Hepatol (2016) 64(1 Suppl):S84–S101. doi: 10.1016/j.jhep.2016.02.021 27084040

[81] Perugorria MJ Olaizola P Labiano I Esparza-Baquer A Marzioni M Marin JJG. Wnt-Beta-Catenin signalling in liver development, health and disease. Nat Rev Gastroenterol Hepatol (2019) 16(2):121–36. doi: 10.1038/s41575-018-0075-9 30451972

[82] Khemlina G Ikeda S Kurzrock R. The biology of hepatocellular carcinoma: Implications for genomic and immune therapies. Mol Cancer (2017) 16(1):149. doi: 10.1186/s12943-017-0712-x 28854942 PMC5577674

[83] Lu C Rong D Zhang B Zheng W Wang X Chen Z. Current perspectives on the immunosuppressive tumor microenvironment in hepatocellular carcinoma: Challenges and opportunities. Mol Cancer (2019) 18(1):130. doi: 10.1186/s12943-019-1047-6 31464625 PMC6714090

[84] Bozward AG Warricker F Oo YH Khakoo SI. Natural killer cells and regulatory T cells cross talk in hepatocellular carcinoma: Exploring therapeutic options for the next decade. Front Immunol (2021) 12:643310. doi: 10.3389/fimmu.2021.643310 33995362 PMC8120158

[85] Singh V Khurana A Allawadhi P Banothu AK Bharani KK Weiskirchen R. Emerging role of pd-1/Pd-L1 inhibitors in chronic liver diseases. Front Pharmacol (2021) 12:790963. doi: 10.3389/fphar.2021.790963 35002724 PMC8733625

[86] Chen J Gingold JA Su X. Immunomodulatory tgf-beta signaling in hepatocellular carcinoma. Trends Mol Med (2019) 25(11):1010–23. doi: 10.1016/j.molmed.2019.06.007 31353124

[87] An L Zhang J Feng D Zhao Y Ouyang W Shi R. Kif2c is a novel prognostic biomarker and correlated with immune infiltration in endometrial cancer. Stem Cells Int (2021) 2021:1434856. doi: 10.1155/2021/1434856 34650608 PMC8510809

[88] Wei S Dai M Zhang C Teng K Wang F Li H. Kif2c: A novel link between Wnt/Beta-catenin and Mtorc1 signaling in the pathogenesis of hepatocellular carcinoma. Protein Cell (2021) 12(10):788–809. doi: 10.1007/s13238-020-00766-y 32748349 PMC8464548

[89] Valvezan AJ McNamara MC Miller SK Torrence ME Asara JM Henske EP. Impdh inhibitors for antitumor therapy in tuberous sclerosis complex. JCI Insight (2020) 5(7):e135071. doi: 10.1172/jci.insight.135071 PMC7205253 32271165

[90] Evison BJ Sleebs BE Watson KG Phillips DR Cutts SM. Mitoxantrone, more than just another topoisomerase ii poison. Med Res Rev (2016) 36(2):248–99. doi: 10.1002/med.21364 26286294

[91] Basch EM Scholz M de Bono JS Vogelzang N de Souza P Marx G. Cabozantinib versus mitoxantrone-prednisone in symptomatic metastatic castration-resistant prostate cancer: A randomized phase 3 trial with a primary pain endpoint. Eur Urol (2019) 75(6):929–37. doi: 10.1016/j.eururo.2018.11.033 PMC6876845 30528222

[92] Guan Y Jiang S Ye W Ren X Wang X Zhang Y. Combined treatment of mitoxantrone sensitizes breast cancer cells to rapalogs through blocking eef-2k-Mediated activation of akt and autophagy. Cell Death Dis (2020) 11(11):948. doi: 10.1038/s41419-020-03153-x 33144562 PMC7642277

[93] Zeidner JF Lin TL Vigil CE Fine G Yair Levy M Nazha A. A prospective biomarker analysis of alvocidib followed by cytarabine and mitoxantrone in mcl-1-Dependent Relapsed/Refractory acute myeloid leukemia. Blood Cancer J (2021) 11(10):175. doi: 10.1038/s41408-021-00568-3 34718324 PMC8557202

[94] Ge C Wang Y Feng Y Wang S Zhang K Xu X. Suppression of oxidative phosphorylation and Idh2 sensitizes colorectal cancer to a naphthalimide derivative and mitoxantrone. Cancer Lett (2021) 519:30–45. doi: 10.1016/j.canlet.2021.06.015 34166768

[95] Orlando BJ Liao M. Abcg2 transports anticancer drugs *via* a closed-to-Open switch. Nat Commun (2020) 11(1):2264. doi: 10.1038/s41467-020-16155-2 32385283 PMC7210939

[96] Advani AS Cooper B Visconte V Elson P Chan R Carew J. A phase I/Ii trial of mec (Mitoxantrone, etoposide, cytarabine) in combination with ixazomib for relapsed refractory acute myeloid leukemia. Clin Cancer Res (2019) 25(14):4231–7. doi: 10.1158/1078-0432.CCR-18-3886 PMC6635077 30992301

[97] Kreft D Wang Y Rattay M Toensing K Anselmetti D. Binding mechanism of anti-cancer chemotherapeutic drug mitoxantrone to DNA characterized by magnetic tweezers. J Nanobiotechnol (2018) 16(1):56. doi: 10.1186/s12951-018-0381-y PMC6043947 30005668

