# Supplementary material for: Hallmark-guided subtypes of hepatocellular carcinoma for the identification of immune-related gene classifiers in the prediction of prognosis, treatment efficacy, and drug candidates
Source: Front Immunol. 2022 Aug 10;13:958161. doi: 10.3389/fimmu.2022.958161 (PMC9399518; doi:10.3389/fimmu.2022.958161)
Supplement: Supplementary file 1 [file DataSheet_1.docx]

Supplementary Material

# Supplementary Table 1 Demographic information of HCC patients in the current study.

| **Variables** | **TCGA-training set** | **TCGA-validation set** | **Whole TCGA cohort** | **ICGC-LIRI-JP set** |
| --- | --- | --- | --- | --- |
| **Total** | 222 | 114 | 336 | 238 |
| **Gender** |  |  |  |  |
| Female | 72 | 36 | 108 | 61 |
| Male | 150 | 78 | 228 | 177 |
| **Age** |  |  |  |  |
| <60 | 94 | 59 | 153 | 49 |
| ≥60 | 128 | 55 | 183 | 189 |
| **Race** |  |  |  |  |
| White | 111 | 52 | 163 | - |
| Others | 104 | 59 | 163 | - |
| **BMI** |  |  |  |  |
| <25 | 100 | 59 | 159 | - |
| ≥25 | 103 | 47 | 150 | - |
| **TNM stage** |  |  |  |  |
| I | 103 | 54 | 157 | 36 |
| II | 52 | 25 | 77 | 109 |
| III | 55 | 24 | 79 | 72 |
| VI | 0 | 4 | 4 | 21 |
| **Grade** |  |  |  |  |
| G1 | 32 | 20 | 52 | - |
| G2 | 100 | 57 | 157 | - |
| G3 | 77 | 33 | 110 | - |
| G4 | 9 | 3 | 12 | - |
| NA | 4 | 1 | 5 | - |
| **Progression** |  |  |  |  |
| Yes | 114 | 61 | 161 | 197 |
| No | 108 | 53 | 175 | 41 |
| **Tumor burden** |  |  |  |  |
| Tumor free | 137 | 70 | 207 | - |
| With tumor | 74 | 34 | 108 | - |

# Supplementary Table 2 67 IRDEGs between the two HCC subtypes.

| **Gene symbol** | **logFC** | **AveExpr** | ***t*** | ***P*.Value** | **adj.P.Val** | **B** |
| --- | --- | --- | --- | --- | --- | --- |
| SAA1 | -2.598245178 | 8.83104197 | -7.379711465 | 1.25E-12 | 4.91E-12 | 17.76427483 |
| AQP9 | -2.558267643 | 7.392620179 | -10.188765 | 2.00E-21 | 2.33E-20 | 37.74498975 |
| SAA2 | -2.283036728 | 5.610595983 | -6.84895736 | 3.55E-11 | 1.19E-10 | 14.48494325 |
| MASP2 | -2.013637403 | 6.432444167 | -10.38116673 | 4.37E-22 | 5.50E-21 | 39.24863872 |
| HRG | -1.922776667 | 9.140875446 | -6.719624665 | 7.80E-11 | 2.52E-10 | 13.71363607 |
| CCL16 | -1.848886349 | 6.374448634 | -7.980111032 | 2.32E-14 | 1.13E-13 | 21.68367296 |
| HFE2 | -1.789056585 | 7.440643671 | -8.540943479 | 4.67E-16 | 2.75E-15 | 25.53130715 |
| LECT2 | -1.605725168 | 5.530921087 | -6.038289237 | 4.12E-09 | 1.11E-08 | 9.839845842 |
| RBP4 | -1.518075399 | 12.9551353 | -9.851913015 | 2.76E-20 | 2.76E-19 | 35.14858048 |
| FABP4 | -1.490726107 | 3.46196341 | -7.743483981 | 1.15E-13 | 5.08E-13 | 20.11341188 |
| NR1I3 | -1.481804071 | 5.128433537 | -7.837867921 | 6.09E-14 | 2.80E-13 | 20.7358398 |
| GCGR | -1.462437769 | 3.490390412 | -5.523649851 | 6.65E-08 | 1.58E-07 | 7.135522459 |
| AZGP1 | -1.410868924 | 9.243132388 | -8.089343768 | 1.10E-14 | 5.54E-14 | 22.41940731 |
| AR | -1.356461553 | 3.833158638 | -8.548155568 | 4.44E-16 | 2.62E-15 | 25.58189635 |
| KNG1 | -1.294830666 | 10.48152938 | -6.852602641 | 3.47E-11 | 1.16E-10 | 14.50684394 |
| MBL2 | -1.274138535 | 5.262646079 | -5.920139426 | 7.93E-09 | 2.07E-08 | 9.201642267 |
| NR1I2 | -1.228656359 | 3.647412467 | -6.701093898 | 8.72E-11 | 2.80E-10 | 13.60403978 |
| FGA | -1.218762651 | 12.49635004 | -6.911669634 | 2.41E-11 | 8.23E-11 | 14.86293821 |
| ANGPTL3 | -1.205005254 | 7.789060127 | -6.687027802 | 9.49E-11 | 3.04E-10 | 13.52100317 |
| TFR2 | -1.147743004 | 8.455761268 | -7.496451034 | 5.86E-13 | 2.38E-12 | 18.50939533 |
| PLA2G2A | -1.136257178 | 5.733754638 | -2.724483256 | 0.006776567 | 0.00927232 | -3.791388986 |
| KLKB1 | -1.112107479 | 6.137729352 | -8.350372014 | 1.80E-15 | 9.90E-15 | 24.20457313 |
| LEAP2 | -1.11169756 | 6.528863465 | -7.016834401 | 1.25E-11 | 4.44E-11 | 15.50260053 |
| PGLYRP2 | -1.099500678 | 6.078994703 | -4.615645083 | 5.58E-06 | 1.08E-05 | 2.863306378 |
| GREM2 | -1.088758717 | 2.466435736 | -4.82754979 | 2.10E-06 | 4.24E-06 | 3.801187898 |
| TMPRSS6 | -1.088727538 | 6.879526454 | -6.355426037 | 6.76E-10 | 1.98E-09 | 11.60258135 |
| IL27 | -1.074595815 | 3.479943652 | -7.838097768 | 6.08E-14 | 2.79E-13 | 20.73736193 |
| CD14 | -1.074396867 | 8.773533023 | -8.525985279 | 5.20E-16 | 3.04E-15 | 25.42647032 |
| RBP5 | -1.060172691 | 7.257418491 | -6.41506054 | 4.77E-10 | 1.42E-09 | 11.94198907 |
| HAMP | -1.057233799 | 2.94936252 | -3.82699677 | 0.00015463 | 0.000254893 | -0.29570828 |
| OSGIN1 | -1.009099289 | 7.138391795 | -6.260342774 | 1.17E-09 | 3.34E-09 | 11.06657631 |
| ORM1 | -1.000717645 | 12.37404524 | -5.363622627 | 1.52E-07 | 3.47E-07 | 6.335350819 |
| PTHLH | 1.004313923 | 0.98754781 | 7.533270187 | 4.61E-13 | 1.90E-12 | 18.74613428 |
| S100A9 | 1.016391889 | 5.258369268 | 4.8918136 | 1.55E-06 | 3.17E-06 | 4.092863634 |
| PI3 | 1.017006867 | 2.259169426 | 4.340759716 | 1.88E-05 | 3.43E-05 | 1.702144574 |
| VCAM1 | 1.018683746 | 3.603180003 | 5.732045758 | 2.21E-08 | 5.52E-08 | 8.206811075 |
| LIF | 1.021670178 | 1.838420366 | 6.531810892 | 2.40E-10 | 7.34E-10 | 12.6136177 |
| ADM2 | 1.025244078 | 3.370156164 | 6.830397261 | 3.97E-11 | 1.32E-10 | 14.37357138 |
| TFRC | 1.036351055 | 4.971502167 | 10.00725888 | 8.26E-21 | 8.82E-20 | 36.3401206 |
| ROBO1 | 1.08228459 | 3.799278089 | 6.287834948 | 9.99E-10 | 2.87E-09 | 11.22090108 |
| CXCL8 | 1.098942695 | 3.08720747 | 4.693851969 | 3.91E-06 | 7.66E-06 | 3.205153792 |
| EPO | 1.1055807 | 1.579265482 | 5.531730403 | 6.38E-08 | 1.52E-07 | 7.176448098 |
| FGFR3 | 1.106399157 | 5.238190971 | 7.797272777 | 7.99E-14 | 3.62E-13 | 20.46748902 |
| TMSB10 | 1.122871328 | 11.09317168 | 7.758970787 | 1.03E-13 | 4.60E-13 | 20.21518273 |
| FGFR2 | 1.128944777 | 3.639571461 | 4.658236617 | 4.60E-06 | 8.95E-06 | 3.04885104 |
| NTS | 1.1691141 | 1.511021194 | 4.428695461 | 1.28E-05 | 2.38E-05 | 2.066712286 |
| S100A11 | 1.202411177 | 7.674287176 | 7.693440249 | 1.60E-13 | 6.97E-13 | 19.78552162 |
| PLXNA1 | 1.209296316 | 2.688445605 | 12.53787106 | 7.47E-30 | 2.40E-28 | 56.97150051 |
| TNFRSF21 | 1.239022654 | 4.331957446 | 8.2247424 | 4.32E-15 | 2.28E-14 | 23.34069534 |
| CXCL1 | 1.243025883 | 2.625401969 | 4.952289103 | 1.16E-06 | 2.41E-06 | 4.370396355 |
| S100A14 | 1.245135869 | 4.409930421 | 4.983883287 | 9.99E-07 | 2.09E-06 | 4.516558851 |
| CXCL5 | 1.252685892 | 1.141437454 | 6.230604904 | 1.39E-09 | 3.94E-09 | 10.90024782 |
| CDK4 | 1.272371143 | 5.357266096 | 15.49527619 | 2.97E-41 | 2.79E-39 | 83.03372894 |
| MMP12 | 1.296820144 | 1.158615712 | 7.92381853 | 3.40E-14 | 1.62E-13 | 21.30717346 |
| VEGFB | 1.325676728 | 5.985772854 | 8.116948564 | 9.10E-15 | 4.62E-14 | 22.60640628 |
| IKBKE | 1.33287911 | 2.517818326 | 12.14071489 | 2.23E-28 | 6.03E-27 | 53.6033887 |
| NCK2 | 1.355605807 | 4.135580321 | 9.395271231 | 8.96E-19 | 7.34E-18 | 31.70670628 |
| SRC | 1.389977594 | 3.699253563 | 10.29532813 | 8.62E-22 | 1.06E-20 | 38.57599199 |
| MDK | 1.393177888 | 7.037078875 | 7.21793228 | 3.53E-12 | 1.32E-11 | 16.74566245 |
| NDRG1 | 1.417493236 | 5.782982143 | 10.78997749 | 1.65E-23 | 2.49E-22 | 42.49059898 |
| CLDN4 | 1.470060994 | 3.027405426 | 5.615842462 | 4.10E-08 | 9.93E-08 | 7.6054018 |
| CCL20 | 1.477377651 | 5.042148728 | 5.701748315 | 2.60E-08 | 6.44E-08 | 8.049028751 |
| MMP9 | 1.525780294 | 3.491125936 | 7.610432291 | 2.77E-13 | 1.17E-12 | 19.24493547 |
| S100P | 1.52691967 | 3.803708757 | 4.575631621 | 6.69E-06 | 1.28E-05 | 2.690360576 |
| DKK1 | 1.540012514 | 1.903653924 | 6.183517611 | 1.82E-09 | 5.09E-09 | 10.63816634 |
| SPP1 | 2.189651006 | 7.09763371 | 6.019484271 | 4.57E-09 | 1.23E-08 | 9.737587791 |
| BIRC5 | 2.208650949 | 3.933255532 | 19.00614225 | 3.10E-55 | 1.44E-52 | 115.0174646 |

# Supplementary Table 3 UniCox analysis of the 67 IRDEGs for HCC OS.

| **Gene symbol** | **HR** | ***P*** | **lower** | **upper** |
| --- | --- | --- | --- | --- |
| SPP1 | 1.129308509 | 0.000467545 | 1.05493825 | 1.208921668 |
| PLXNA1 | 1.43280554 | 0.001009021 | 1.156337522 | 1.775374125 |
| EPO | 1.192871396 | 0.001107817 | 1.072919248 | 1.326234168 |
| CDK4 | 1.493259106 | 0.001212068 | 1.171296055 | 1.903722589 |
| S100A9 | 1.185934777 | 0.001589054 | 1.066828912 | 1.31833819 |
| BIRC5 | 1.2935461 | 0.001720469 | 1.101260047 | 1.519406355 |
| TMPRSS6 | 0.828164584 | 0.003080997 | 0.730952494 | 0.938305272 |
| LEAP2 | 0.816739391 | 0.003179299 | 0.713952396 | 0.934324525 |
| ROBO1 | 1.208171866 | 0.008899954 | 1.048559606 | 1.392080384 |
| KLKB1 | 0.804277356 | 0.008946543 | 0.683096507 | 0.946955603 |
| IL27 | 0.811046123 | 0.009647184 | 0.69210061 | 0.950433802 |
| CXCL5 | 1.135490914 | 0.010798559 | 1.029804143 | 1.25202411 |
| FGA | 0.849191094 | 0.011697602 | 0.747848055 | 0.964267421 |
| IKBKE | 1.245726562 | 0.017161477 | 1.039793851 | 1.492444551 |
| MASP2 | 0.889586222 | 0.019561799 | 0.806363447 | 0.981398213 |
| AQP9 | 0.908087878 | 0.019630015 | 0.837444679 | 0.984690232 |
| S100A11 | 1.177109515 | 0.020385767 | 1.025577627 | 1.351030653 |
| LECT2 | 0.906931368 | 0.021677851 | 0.834367904 | 0.985805545 |
| TNFRSF21 | 1.187875442 | 0.021776153 | 1.025402957 | 1.376091279 |
| NCK2 | 1.199789096 | 0.022179836 | 1.026407816 | 1.40245802 |
| FABP4 | 0.864485 | 0.024497884 | 0.761462167 | 0.981446416 |
| CCL20 | 1.108843329 | 0.026986695 | 1.011829448 | 1.215158869 |
| NDRG1 | 1.21278456 | 0.027389447 | 1.021743299 | 1.439545912 |
| KNG1 | 0.886425079 | 0.028882531 | 0.795569887 | 0.98765606 |
| RBP4 | 0.860835029 | 0.030341243 | 0.75165711 | 0.985871 |
| HFE2 | 0.900224542 | 0.031986679 | 0.817770319 | 0.990992468 |
| CXCL8 | 1.113889489 | 0.032169456 | 1.009222238 | 1.229411866 |
| TFRC | 1.283404674 | 0.035801118 | 1.016685308 | 1.620095761 |
| CXCL1 | 1.088274999 | 0.061877754 | 0.99580474 | 1.18933203 |
| HRG | 0.929923853 | 0.066432133 | 0.860508479 | 1.004938818 |
| RBP5 | 0.879161598 | 0.07019547 | 0.764759699 | 1.010677101 |
| TFR2 | 0.876341309 | 0.078240978 | 0.756604713 | 1.015026838 |
| CD14 | 0.844436205 | 0.078585711 | 0.699426013 | 1.019510987 |
| CLDN4 | 1.083704505 | 0.079325881 | 0.990636127 | 1.185516479 |
| MMP9 | 1.097786021 | 0.080003895 | 0.988908041 | 1.218651379 |
| PTHLH | 1.157843257 | 0.080876476 | 0.982165319 | 1.364944355 |
| LIF | 1.134215287 | 0.081745156 | 0.984257141 | 1.307020557 |
| AZGP1 | 0.894442057 | 0.103388395 | 0.782075255 | 1.022953468 |
| MMP12 | 1.092136221 | 0.127742834 | 0.975034653 | 1.223301676 |
| AR | 0.900880297 | 0.145938842 | 0.782636915 | 1.036988281 |
| NR1I2 | 0.918954261 | 0.186205212 | 0.810717909 | 1.041640902 |
| TMSB10 | 1.102306852 | 0.186709705 | 0.953912867 | 1.273785518 |
| S100A14 | 1.065107244 | 0.192025166 | 0.968811662 | 1.17097418 |
| MDK | 1.083066625 | 0.193827854 | 0.960240872 | 1.22160319 |
| SRC | 1.119050846 | 0.194530934 | 0.944162845 | 1.326333483 |
| VCAM1 | 1.081221217 | 0.257121642 | 0.944620927 | 1.237575082 |
| ADM2 | 1.096054225 | 0.259457101 | 0.934548142 | 1.285471353 |
| ANGPTL3 | 0.935562476 | 0.275757489 | 0.829952149 | 1.054611579 |
| HAMP | 0.953135799 | 0.298171787 | 0.870730888 | 1.043339411 |
| PGLYRP2 | 0.94806391 | 0.304458311 | 0.856308431 | 1.049651206 |
| NTS | 1.038238073 | 0.336487628 | 0.961753295 | 1.120805409 |
| CCL16 | 0.956459604 | 0.35805673 | 0.869836092 | 1.051709606 |
| DKK1 | 1.040902083 | 0.35894572 | 0.955462959 | 1.133981319 |
| VEGFB | 1.059196941 | 0.433164716 | 0.917316392 | 1.223022034 |
| FGFR2 | 1.039621509 | 0.445369846 | 0.940883129 | 1.148721715 |
| GREM2 | 0.9618291 | 0.489317387 | 0.861358477 | 1.074018822 |
| SAA1 | 0.97670932 | 0.497138555 | 0.912478112 | 1.045461895 |
| S100P | 1.01991941 | 0.568350645 | 0.953096276 | 1.091427623 |
| ORM1 | 0.959021122 | 0.57385803 | 0.828890104 | 1.109581967 |
| NR1I3 | 0.971762024 | 0.635318703 | 0.86327358 | 1.093884318 |
| MBL2 | 0.979459242 | 0.727604602 | 0.871498395 | 1.100794231 |
| PLA2G2A | 0.98936136 | 0.73325217 | 0.930338777 | 1.052128456 |
| OSGIN1 | 0.974808405 | 0.754388436 | 0.830810031 | 1.14376499 |
| GCGR | 0.987977749 | 0.787444189 | 0.904823713 | 1.07877371 |
| FGFR3 | 1.017009428 | 0.839858619 | 0.863531875 | 1.197764907 |
| SAA2 | 0.995061769 | 0.897516808 | 0.922855237 | 1.072917923 |
| PI3 | 0.996085268 | 0.936111774 | 0.904990661 | 1.096349282 |

# Supplementary Table 4 UniCox analysis of TME cells for HCC OS.

| **TMEcell** | **HR** | ***P*** | **lower** | **upper** |
| --- | --- | --- | --- | --- |
| Eosinophil | 0.006042988 | 0.005068333 | 0.00016968 | 0.215215663 |
| Effector memeory CD8 T cell | 0.020373533 | 0.005874459 | 0.001276361 | 0.32520646 |
| Endothelial cells | 0.08080134 | 0.008569466 | 0.012382516 | 0.527264132 |
| Natural killer T cell | 372.0802267 | 0.009170033 | 4.335198814 | 31934.79723 |
| Activated CD8 T cell | 0.051938162 | 0.010861819 | 0.005334251 | 0.505707825 |
| Activated B cell | 0.064212867 | 0.020408636 | 0.006305847 | 0.653883964 |
| Type 1 T helper cell | 0.0037373 | 0.026311748 | 2.70E-05 | 0.517776165 |
| Immature dendritic cell | 633.0392579 | 0.049669578 | 1.009359263 | 397022.8606 |
| Activated CD4 T cell | 6.536277877 | 0.054475704 | 0.964589736 | 44.29129494 |
| Effector memeory CD4 T cell | 97.58773586 | 0.112373035 | 0.34152518 | 27884.81418 |
| Plasmacytoid dendritic cell | 100.7992895 | 0.126617142 | 0.270966846 | 37497.19533 |
| Activated dendritic cell | 8.513320795 | 0.309628003 | 0.136726165 | 530.086038 |
| Immature B cell | 0.271820359 | 0.335564223 | 0.019180233 | 3.852211101 |
| Memory B cell | 0.227144342 | 0.42709416 | 0.005857355 | 8.808506425 |
| Type 17 T helper cell | 4.837953876 | 0.464422479 | 0.070860777 | 330.3068178 |
| CD56dim natural killer cell | 0.133140263 | 0.465668263 | 0.000591177 | 29.98481212 |
| Monocyte | 9.541915862 | 0.501419451 | 0.013289672 | 6851.046275 |
| Fibroblasts | 0.710080058 | 0.560561674 | 0.224166607 | 2.249280994 |
| CD56bright natural killer cell | 0.14024313 | 0.623974312 | 5.45E-05 | 361.1790633 |
| Macrophage | 0.512203097 | 0.655916024 | 0.026996678 | 9.717937001 |
| Mast cell | 0.607658349 | 0.673818279 | 0.059742009 | 6.180720633 |
| Natural killer cell | 0.518166572 | 0.76385537 | 0.007106515 | 37.78175203 |
| Gamma delta T cell | 2.245269978 | 0.781156678 | 0.007465664 | 675.2564272 |
| Regulatory T cell | 1.308328831 | 0.806527742 | 0.152281678 | 11.24051398 |
| Type 2 T helper cell | 1.621675456 | 0.815227585 | 0.02811721 | 93.5310171 |
| T follicular helper cell | 0.657381855 | 0.820250807 | 0.017634315 | 24.50624889 |
| MDSC | 1.19870681 | 0.842714114 | 0.200082658 | 7.181522042 |
| Central memory CD8 T cell | 1.573920159 | 0.896937333 | 0.001645872 | 1505.113962 |
| Neutrophil | 0.909038992 | 0.938620187 | 0.080241003 | 10.29837436 |
| Central memory CD4 T cell | 0.925435796 | 0.974183423 | 0.008475147 | 101.0520955 |

# Supplementary Table 5 170 DEGs between the HGSIS high- and low-risk groups of HCC.

| **Gene symbol** | **logFC** | **AveExpr** | ***t*** | ***P*.Value** | **adj.P.Val** | **B** |
| --- | --- | --- | --- | --- | --- | --- |
| LPCAT1 | 1.641943075 | 4.034857973 | 16.26701295 | 2.62E-44 | 4.37E-40 | 89.90137367 |
| G6PD | 2.018217866 | 4.561568372 | 15.49358636 | 3.01E-41 | 2.52E-37 | 82.94733405 |
| SPP1 | 4.436788153 | 7.09763371 | 15.12918367 | 8.19E-40 | 4.56E-36 | 79.68862613 |
| PKM | 2.061716736 | 5.310079735 | 14.73339049 | 2.91E-38 | 9.72E-35 | 76.16532723 |
| SLC1A5 | 1.962797791 | 3.946154214 | 14.10075499 | 8.40E-36 | 2.34E-32 | 70.57544856 |
| HMGA1 | 1.51361717 | 6.51755753 | 13.38331775 | 4.80E-33 | 4.71E-30 | 64.31221037 |
| KIF2C | 1.648956194 | 2.905574671 | 13.21884938 | 2.03E-32 | 1.46E-29 | 62.88966614 |
| IL4I1 | 1.602029158 | 2.055067379 | 13.15027463 | 3.69E-32 | 2.17E-29 | 62.29813827 |
| MYBL2 | 2.182292741 | 3.950260057 | 13.1478916 | 3.77E-32 | 2.17E-29 | 62.27759941 |
| SLC16A3 | 1.620897696 | 2.560930776 | 13.07212911 | 7.30E-32 | 3.94E-29 | 61.62523301 |
| CDC20 | 1.886348543 | 4.322459247 | 12.50085757 | 1.03E-29 | 3.36E-27 | 56.74678948 |
| PLK1 | 1.532192978 | 2.797412668 | 12.29777569 | 5.85E-29 | 1.41E-26 | 55.03112203 |
| S100A11 | 1.718731257 | 7.674287176 | 12.29576552 | 5.95E-29 | 1.42E-26 | 55.01419174 |
| CENPM | 1.558782389 | 3.335166063 | 12.21312806 | 1.20E-28 | 2.48E-26 | 54.31909187 |
| CCNB1 | 1.546322304 | 4.473881685 | 12.13094952 | 2.42E-28 | 4.70E-26 | 53.62962012 |
| SLC38A1 | 1.787921168 | 3.152746019 | 11.9328059 | 1.30E-27 | 2.10E-25 | 51.97466942 |
| SLC10A1 | -3.078468619 | 6.198408378 | -11.8479495 | 2.65E-27 | 3.75E-25 | 51.26924224 |
| SLC27A5 | -2.117284457 | 6.495409917 | -11.74766132 | 6.16E-27 | 8.10E-25 | 50.43816808 |
| SOX4 | 1.7982493 | 3.722911893 | 11.49266065 | 5.19E-26 | 5.55E-24 | 48.3383257 |
| TREM2 | 1.586808192 | 3.491769689 | 11.34038451 | 1.83E-25 | 1.65E-23 | 47.09384079 |
| EGLN3 | 1.510583 | 1.776968233 | 11.28683966 | 2.85E-25 | 2.44E-23 | 46.65797918 |
| BIRC5 | 1.587928702 | 3.933255532 | 11.19748085 | 5.95E-25 | 4.80E-23 | 45.93264188 |
| LIF | 1.566636156 | 1.838420366 | 11.10990355 | 1.22E-24 | 9.11E-23 | 45.22429832 |
| TNFRSF21 | 1.541894721 | 4.331957446 | 10.95607606 | 4.29E-24 | 2.67E-22 | 43.98631907 |
| SPHK1 | 2.006786609 | 2.713841847 | 10.94475406 | 4.71E-24 | 2.91E-22 | 43.89551955 |
| CTHRC1 | 1.875133059 | 2.839495474 | 10.80465696 | 1.47E-23 | 8.16E-22 | 42.77565947 |
| PLBD1 | 1.577760518 | 2.671196491 | 10.748771 | 2.30E-23 | 1.23E-21 | 42.33086557 |
| IER3 | 1.640934591 | 5.602393616 | 10.73878243 | 2.50E-23 | 1.33E-21 | 42.25148435 |
| CA9 | 2.446997271 | 1.847039158 | 10.58586363 | 8.54E-23 | 4.11E-21 | 41.04071676 |
| UBE2C | 1.672577479 | 4.691046537 | 10.54423264 | 1.19E-22 | 5.46E-21 | 40.71257992 |
| PKIB | 1.62918884 | 2.19142158 | 10.52938233 | 1.34E-22 | 6.05E-21 | 40.5956852 |
| GLYATL1 | -1.803322671 | 4.843921304 | -10.51264262 | 1.53E-22 | 6.85E-21 | 40.46401673 |
| SEL1L3 | 1.698190494 | 3.230704609 | 10.51090499 | 1.55E-22 | 6.92E-21 | 40.45035522 |
| RGS2 | 1.59415974 | 4.073708387 | 10.48682427 | 1.88E-22 | 8.26E-21 | 40.26114503 |
| C12orf75 | 1.809545902 | 3.152965316 | 10.47576969 | 2.06E-22 | 8.95E-21 | 40.17435856 |
| MMP9 | 1.951120878 | 3.491125936 | 10.40938646 | 3.49E-22 | 1.45E-20 | 39.65417537 |
| UPB1 | -1.811069794 | 5.81607032 | -10.39972686 | 3.77E-22 | 1.54E-20 | 39.57862199 |
| CAPG | 1.589544793 | 4.73588358 | 10.3433656 | 5.89E-22 | 2.34E-20 | 39.1385016 |
| PHLDA2 | 1.652345726 | 3.875651371 | 10.26263656 | 1.12E-21 | 4.20E-20 | 38.51023906 |
| TRNP1 | 1.999914058 | 3.926836367 | 10.21514158 | 1.62E-21 | 5.87E-20 | 38.14180919 |
| EPO | 1.849223906 | 1.579265482 | 10.21080409 | 1.68E-21 | 6.02E-20 | 38.1082067 |
| TAT | -2.884636118 | 7.195537361 | -10.168306 | 2.34E-21 | 8.06E-20 | 37.77936948 |
| TOP2A | 1.607093578 | 4.027768547 | 10.16818338 | 2.35E-21 | 8.06E-20 | 37.7784217 |
| TTC36 | -2.416164065 | 3.656602816 | -10.10526836 | 3.84E-21 | 1.27E-19 | 37.29293415 |
| ACSM2A | -1.799783149 | 5.586080226 | -10.09997436 | 4.00E-21 | 1.31E-19 | 37.25215538 |
| SERPINC1 | -2.263085082 | 11.5002438 | -10.08356664 | 4.55E-21 | 1.47E-19 | 37.12584165 |
| MMP12 | 1.563436755 | 1.158615712 | 10.07738291 | 4.78E-21 | 1.53E-19 | 37.078265 |
| GYS2 | -2.158702649 | 4.315698036 | -10.07411415 | 4.90E-21 | 1.56E-19 | 37.05312186 |
| SLC6A8 | 1.796244468 | 3.075645964 | 10.03284393 | 6.76E-21 | 2.08E-19 | 36.73604855 |
| SEC14L2 | -1.613405103 | 5.63109381 | -9.977829885 | 1.04E-20 | 3.05E-19 | 36.31446869 |
| GNAZ | 1.542001319 | 2.869914809 | 9.960970489 | 1.18E-20 | 3.44E-19 | 36.1855229 |
| RGS1 | 1.624724892 | 3.236001203 | 9.818825558 | 3.56E-20 | 9.35E-19 | 35.10307755 |
| GNMT | -2.254334895 | 6.350104259 | -9.78086827 | 4.76E-20 | 1.22E-18 | 34.81547469 |
| ACSM2B | -1.65104497 | 6.111991358 | -9.752350833 | 5.93E-20 | 1.47E-18 | 34.59980332 |
| S100A9 | 1.829729492 | 5.258369268 | 9.692481594 | 9.39E-20 | 2.23E-18 | 34.14816544 |
| HKDC1 | 1.777337818 | 4.240177704 | 9.624293467 | 1.58E-19 | 3.64E-18 | 33.63567128 |
| SPP2 | -2.445673072 | 6.329606687 | -9.569551555 | 2.40E-19 | 5.35E-18 | 33.2257164 |
| DSG2 | 1.56947166 | 3.802843241 | 9.563207143 | 2.52E-19 | 5.57E-18 | 33.17828975 |
| SELM | 1.624503033 | 4.466238425 | 9.519577481 | 3.50E-19 | 7.51E-18 | 32.85262879 |
| CFHR4 | -1.925055786 | 4.374590832 | -9.509094852 | 3.79E-19 | 8.08E-18 | 32.77451103 |
| ASPDH | -1.852608735 | 5.447381697 | -9.490012058 | 4.38E-19 | 9.23E-18 | 32.63243045 |
| PITX1 | 1.61699948 | 1.600895199 | 9.490124891 | 4.38E-19 | 9.23E-18 | 32.63327006 |
| CD24 | 2.418607909 | 5.756614292 | 9.472690673 | 5.00E-19 | 1.04E-17 | 32.50360629 |
| CYP4A22 | -1.735285323 | 5.255939392 | -9.444299667 | 6.19E-19 | 1.27E-17 | 32.29274727 |
| PON1 | -1.939679892 | 7.598756005 | -9.430931116 | 6.85E-19 | 1.39E-17 | 32.19358599 |
| CDO1 | -1.710640042 | 7.400738001 | -9.413299817 | 7.82E-19 | 1.56E-17 | 32.06293002 |
| AZGP1 | -1.570889833 | 9.243132388 | -9.30724698 | 1.73E-18 | 3.20E-17 | 31.28003491 |
| S100A6 | 1.564986363 | 7.182961179 | 9.298499925 | 1.85E-18 | 3.39E-17 | 31.2156948 |
| LYPD1 | 1.611987549 | 2.214762368 | 9.29204723 | 1.94E-18 | 3.54E-17 | 31.16825393 |
| G6PC | -1.916521409 | 8.02823274 | -9.173903826 | 4.69E-18 | 7.94E-17 | 30.30309641 |
| KNG1 | -1.642324556 | 10.48152938 | -9.167243638 | 4.92E-18 | 8.32E-17 | 30.25452014 |
| MMP7 | 2.120918643 | 2.339289658 | 9.156815718 | 5.32E-18 | 8.93E-17 | 30.17850611 |
| EPS8L3 | 1.841044252 | 3.102067396 | 9.107336924 | 7.67E-18 | 1.25E-16 | 29.81853812 |
| NXPH4 | 1.575168867 | 1.915898988 | 9.00050678 | 1.69E-17 | 2.60E-16 | 29.04534241 |
| ANXA10 | -1.670847348 | 3.846431388 | -8.997243435 | 1.73E-17 | 2.65E-16 | 29.0218107 |
| APOC3 | -1.914922701 | 12.9977953 | -8.97505201 | 2.03E-17 | 3.10E-16 | 28.86192799 |
| GLYAT | -2.309017246 | 4.768358095 | -8.937822218 | 2.67E-17 | 3.96E-16 | 28.59424026 |
| SLC22A1 | -2.623165666 | 6.078102084 | -8.911506739 | 3.23E-17 | 4.72E-16 | 28.40543961 |
| AGXT | -1.796318482 | 9.045583302 | -8.89578245 | 3.63E-17 | 5.23E-16 | 28.2927889 |
| HAO1 | -1.598986301 | 7.729513231 | -8.87347274 | 4.27E-17 | 6.07E-16 | 28.13316986 |
| MSC | 1.847663893 | 2.901785379 | 8.855395739 | 4.87E-17 | 6.84E-16 | 28.0040161 |
| AQP9 | -2.263958084 | 7.392620179 | -8.806255966 | 6.95E-17 | 9.43E-16 | 27.65375513 |
| BAMBI | 1.553401757 | 5.070210405 | 8.804634274 | 7.04E-17 | 9.54E-16 | 27.6422166 |
| HPX | -1.614592916 | 10.29924552 | -8.741198934 | 1.11E-16 | 1.45E-15 | 27.19190734 |
| CYP8B1 | -2.63064169 | 6.048191365 | -8.733849508 | 1.17E-16 | 1.52E-15 | 27.13986758 |
| IGSF23 | -1.588733743 | 4.376793657 | -8.721369978 | 1.28E-16 | 1.65E-15 | 27.05156555 |
| CYP2C9 | -2.05395652 | 7.470952062 | -8.6697991 | 1.86E-16 | 2.32E-15 | 26.68750586 |
| AP1M2 | 2.127788114 | 2.749465226 | 8.628658479 | 2.50E-16 | 3.02E-15 | 26.39805617 |
| APOA5 | -1.825011941 | 7.638278017 | -8.62798919 | 2.51E-16 | 3.03E-15 | 26.39335452 |
| CYP2A6 | -3.168377892 | 7.122795611 | -8.627382384 | 2.52E-16 | 3.05E-15 | 26.38909201 |
| CYP4A11 | -1.726821215 | 7.124258247 | -8.508294424 | 5.89E-16 | 6.66E-15 | 25.55626232 |
| NR1I3 | -1.575171148 | 5.128433537 | -8.505615041 | 6.01E-16 | 6.77E-15 | 25.53760965 |
| UBD | 1.671307065 | 7.075203098 | 8.496139497 | 6.42E-16 | 7.21E-15 | 25.47167544 |
| HSD17B6 | -1.781219599 | 8.411681988 | -8.440211098 | 9.54E-16 | 1.04E-14 | 25.08347195 |
| BICC1 | 1.567588357 | 2.543865887 | 8.412405427 | 1.16E-15 | 1.25E-14 | 24.89108839 |
| ADH1B | -2.058096581 | 8.303040805 | -8.408470781 | 1.19E-15 | 1.28E-14 | 24.86389841 |
| SPINT1 | 1.991021444 | 2.953344133 | 8.406266441 | 1.21E-15 | 1.30E-14 | 24.84866914 |
| CXCL8 | 1.830011482 | 3.08720747 | 8.385702756 | 1.40E-15 | 1.48E-14 | 24.70672468 |
| F12 | -1.555981487 | 8.575907503 | -8.360096304 | 1.68E-15 | 1.75E-14 | 24.5302884 |
| MASP2 | -1.673716957 | 6.432444167 | -8.301896366 | 2.52E-15 | 2.55E-14 | 24.13058664 |
| CCL20 | 2.031273519 | 5.042148728 | 8.270609774 | 3.14E-15 | 3.14E-14 | 23.91647687 |
| GPLD1 | -1.523807573 | 3.544144264 | -8.268225955 | 3.19E-15 | 3.18E-14 | 23.90018504 |
| FNDC5 | -1.963955649 | 3.286190435 | -8.26333033 | 3.30E-15 | 3.28E-14 | 23.86673639 |
| ETV4 | 1.699120458 | 3.19978035 | 8.253071051 | 3.54E-15 | 3.49E-14 | 23.79668376 |
| ETNPPL | -1.736678071 | 5.079119053 | -8.213129998 | 4.68E-15 | 4.49E-14 | 23.52450582 |
| CXCL5 | 1.571144316 | 1.141437454 | 8.155643772 | 6.96E-15 | 6.49E-14 | 23.13430627 |
| RTP3 | -1.873670646 | 5.100300688 | -8.131954577 | 8.20E-15 | 7.53E-14 | 22.97404277 |
| CYP4F2 | -1.68340449 | 5.774776177 | -8.124522414 | 8.63E-15 | 7.91E-14 | 22.9238265 |
| BHMT | -2.093035778 | 6.76743857 | -8.068841928 | 1.27E-14 | 1.12E-13 | 22.54859483 |
| HPR | -1.784229105 | 8.270088523 | -8.068620345 | 1.27E-14 | 1.12E-13 | 22.54710504 |
| AFM | -1.710887345 | 6.846553803 | -8.003388762 | 1.98E-14 | 1.70E-13 | 22.10972928 |
| CYP2A7 | -2.377704729 | 3.226191268 | -7.976822737 | 2.37E-14 | 2.00E-13 | 21.93229385 |
| ALDOB | -1.944316461 | 11.12761987 | -7.962690319 | 2.61E-14 | 2.18E-13 | 21.83806622 |
| HFE2 | -1.67488927 | 7.440643671 | -7.958017005 | 2.70E-14 | 2.24E-13 | 21.806932 |
| PTGES | 1.543568296 | 2.000452991 | 7.944043666 | 2.97E-14 | 2.45E-13 | 21.71391404 |
| SLC2A2 | -1.530056432 | 7.852989404 | -7.912656584 | 3.67E-14 | 2.98E-13 | 21.50538261 |
| SOX9 | 1.52005744 | 4.107367604 | 7.881393685 | 4.54E-14 | 3.61E-13 | 21.29823763 |
| UCHL1 | 1.583777066 | 1.7692309 | 7.86723538 | 4.99E-14 | 3.94E-13 | 21.20461104 |
| PCK1 | -2.075347262 | 6.90161858 | -7.847385419 | 5.71E-14 | 4.47E-13 | 21.0735412 |
| TTR | -1.701889162 | 10.67267777 | -7.815598344 | 7.07E-14 | 5.42E-13 | 20.86412526 |
| CYP2C8 | -1.985640773 | 7.185545028 | -7.808484113 | 7.41E-14 | 5.67E-13 | 20.81733641 |
| CXCL1 | 1.851637655 | 2.625401969 | 7.793546908 | 8.20E-14 | 6.22E-13 | 20.71919326 |
| CXCL6 | 1.694860605 | 1.88997293 | 7.78322064 | 8.78E-14 | 6.63E-13 | 20.65142172 |
| TESC | 1.734402398 | 3.890120247 | 7.777974763 | 9.10E-14 | 6.85E-13 | 20.61701674 |
| B3GNT3 | 1.69669514 | 3.171279736 | 7.730258518 | 1.25E-13 | 9.19E-13 | 20.30480999 |
| F9 | -1.921460717 | 6.641205341 | -7.709496774 | 1.44E-13 | 1.05E-12 | 20.16938349 |
| FTCD | -1.508550373 | 7.548627579 | -7.670937948 | 1.86E-13 | 1.33E-12 | 19.91854331 |
| PYCR1 | 1.645802035 | 3.459794215 | 7.596365902 | 3.04E-13 | 2.10E-12 | 19.4359264 |
| CCL16 | -1.746905361 | 6.374448634 | -7.526976346 | 4.80E-13 | 3.18E-12 | 18.98983798 |
| GAL3ST1 | 1.596342794 | 2.949799239 | 7.436730137 | 8.65E-13 | 5.46E-12 | 18.41402175 |
| MOGAT2 | -1.56619405 | 3.305672144 | -7.350306564 | 1.51E-12 | 9.13E-12 | 17.86726076 |
| CLDN4 | 1.846510645 | 3.027405426 | 7.313163687 | 1.92E-12 | 1.14E-11 | 17.63369064 |
| ACSM5 | -1.515045063 | 5.199085173 | -7.256036315 | 2.77E-12 | 1.60E-11 | 17.27612313 |
| ADH4 | -2.297315747 | 7.767498596 | -7.091382601 | 7.87E-12 | 4.24E-11 | 16.25700777 |
| CYP2B6 | -1.546814815 | 5.322887079 | -7.062701282 | 9.42E-12 | 5.01E-11 | 16.08124687 |
| LOXL4 | 1.508244242 | 3.627123405 | 7.061645267 | 9.48E-12 | 5.04E-11 | 16.0747856 |
| ADH1C | -2.070915152 | 8.096323092 | -7.040443023 | 1.08E-11 | 5.70E-11 | 15.94521012 |
| CYP3A4 | -2.820972788 | 6.340548356 | -7.032210763 | 1.14E-11 | 5.97E-11 | 15.89497726 |
| HRG | -1.980731244 | 9.140875446 | -7.007780095 | 1.33E-11 | 6.89E-11 | 15.74615936 |
| CFHR3 | -1.617747262 | 5.164025781 | -6.865767304 | 3.20E-11 | 1.55E-10 | 14.88875962 |
| HP | -1.598472398 | 11.03369578 | -6.741098656 | 6.84E-11 | 3.15E-10 | 14.14697993 |
| SULT2A1 | -1.67767335 | 8.706336304 | -6.711257913 | 8.20E-11 | 3.72E-10 | 13.97095752 |
| KRT19 | 1.758072632 | 2.418003972 | 6.700336472 | 8.76E-11 | 3.96E-10 | 13.90668364 |
| OTC | -1.578101874 | 6.711496229 | -6.681164722 | 9.83E-11 | 4.40E-10 | 13.79404897 |
| CPS1 | -1.963390256 | 7.428636398 | -6.662017862 | 1.10E-10 | 4.90E-10 | 13.68180669 |
| RDH16 | -1.572240953 | 6.037058196 | -6.616049306 | 1.45E-10 | 6.33E-10 | 13.41333844 |
| FDCSP | 1.594172186 | 1.405983513 | 6.587860814 | 1.72E-10 | 7.41E-10 | 13.24941655 |
| UGT2B10 | -1.530212226 | 7.254294363 | -6.55403417 | 2.10E-10 | 8.91E-10 | 13.05341929 |
| LECT2 | -1.714626491 | 5.530921087 | -6.549805691 | 2.16E-10 | 9.11E-10 | 13.0289735 |
| S100P | 2.07975705 | 3.803708757 | 6.456548031 | 3.74E-10 | 1.52E-09 | 12.4929375 |
| FETUB | -1.61193264 | 5.910241064 | -6.426107972 | 4.47E-10 | 1.79E-09 | 12.31926431 |
| GCGR | -1.664149429 | 3.490390412 | -6.422463491 | 4.57E-10 | 1.83E-09 | 12.2985139 |
| HPD | -2.048633547 | 8.990498151 | -6.357437703 | 6.68E-10 | 2.61E-09 | 11.9298266 |
| HSD11B1 | -2.007742593 | 7.298438573 | -6.240835483 | 1.31E-09 | 4.86E-09 | 11.27609537 |
| HAO2 | -1.713590424 | 4.602852299 | -6.235561503 | 1.35E-09 | 5.00E-09 | 11.24675252 |
| SFN | 1.627145249 | 4.75473989 | 6.229350442 | 1.40E-09 | 5.16E-09 | 11.21222121 |
| HSD17B13 | -2.128639024 | 4.810626097 | -6.212439738 | 1.54E-09 | 5.65E-09 | 11.11834177 |
| APOA1 | -1.540644069 | 12.97144072 | -6.142552851 | 2.29E-09 | 8.17E-09 | 10.73251617 |
| UROC1 | -1.577083598 | 3.710114049 | -6.023894937 | 4.46E-09 | 1.52E-08 | 10.08542828 |
| GSTA1 | -1.59313904 | 9.745042168 | -5.868681663 | 1.05E-08 | 3.38E-08 | 9.254354927 |
| CYP7A1 | -1.628130785 | 4.845241042 | -5.675354801 | 2.99E-08 | 8.95E-08 | 8.243960788 |
| AFP | 2.038389157 | 4.274964882 | 5.654472803 | 3.34E-08 | 9.93E-08 | 8.13648818 |
| SPINK1 | 2.195224751 | 6.640963072 | 5.641137993 | 3.59E-08 | 1.06E-07 | 8.068029439 |
| SLC25A47 | -1.722562189 | 5.398220379 | -5.501090803 | 7.48E-08 | 2.10E-07 | 7.357148495 |
| NQO1 | 1.690906049 | 4.943147984 | 5.478816886 | 8.40E-08 | 2.35E-07 | 7.245456802 |
| KRT23 | 1.544415622 | 3.147858876 | 5.309691729 | 2.00E-07 | 5.31E-07 | 6.409791054 |
| AKR1B10 | 1.90706161 | 7.744075358 | 5.045788991 | 7.40E-07 | 1.81E-06 | 5.150245043 |
| SDS | -1.654851972 | 6.581411389 | -4.869537502 | 1.72E-06 | 3.98E-06 | 4.339760673 |
| LCN2 | 1.564069349 | 5.979524796 | 4.770892996 | 2.74E-06 | 6.15E-06 | 3.897058625 |
| CRP | 1.571165609 | 8.743778643 | 3.917948579 | 0.000108176 | 0.000194628 | 0.406017297 |

# Supplementary Table 6 Molecular docking results of the top 10 purchasable compounds.

| **Rank** | **ZINC ID** | **Compound name** | **XP score** | **SP score** | **2D-structure** |
| --- | --- | --- | --- | --- | --- |
| 1 | ZINC000012495470 | lactoyl-ph4 | -8.863 | -6.368 | 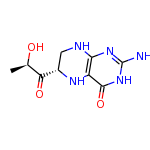 |
| 2 | ZINC000095617488 | dihydrobiopterin | -8.789 | -6.791 | 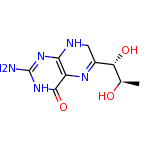 |
| 3 | ZINC000014420733 | 7-biopterin | -8.324 | -7.411 | 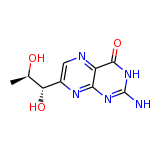 |
| 4 | ZINC000003812887 | mizoribine | -8.316 | -6.328 | 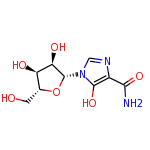 |
| 5 | ZINC000100060558 | trans-3,3',4',5,5',7-hexahydroxyflavanone | -8.272 | -6.438 | 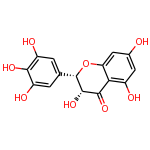 |
| 6 | ZINC000003794794 | mitoxantrone | -8.245 | -6.332 | 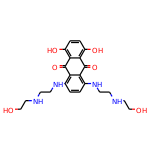 |
| 7 | ZINC000004245708 | imidurea | -8.221 | -6.317 | 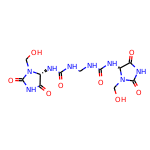 |
| 8 | ZINC000040493557 | succinoadenin | -8.152 | -6.771 | 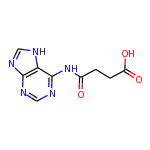 |
| 9 | ZINC000001999315 | dioxethedrin | -8.050 | -6.470 | 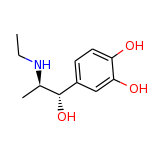 |
| 10 | ZINC000000895903 | 5-hydroxyindoleacetylglycine | -8.037 | -7.049 | 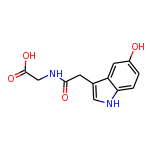 |

**Supplementary Figures**


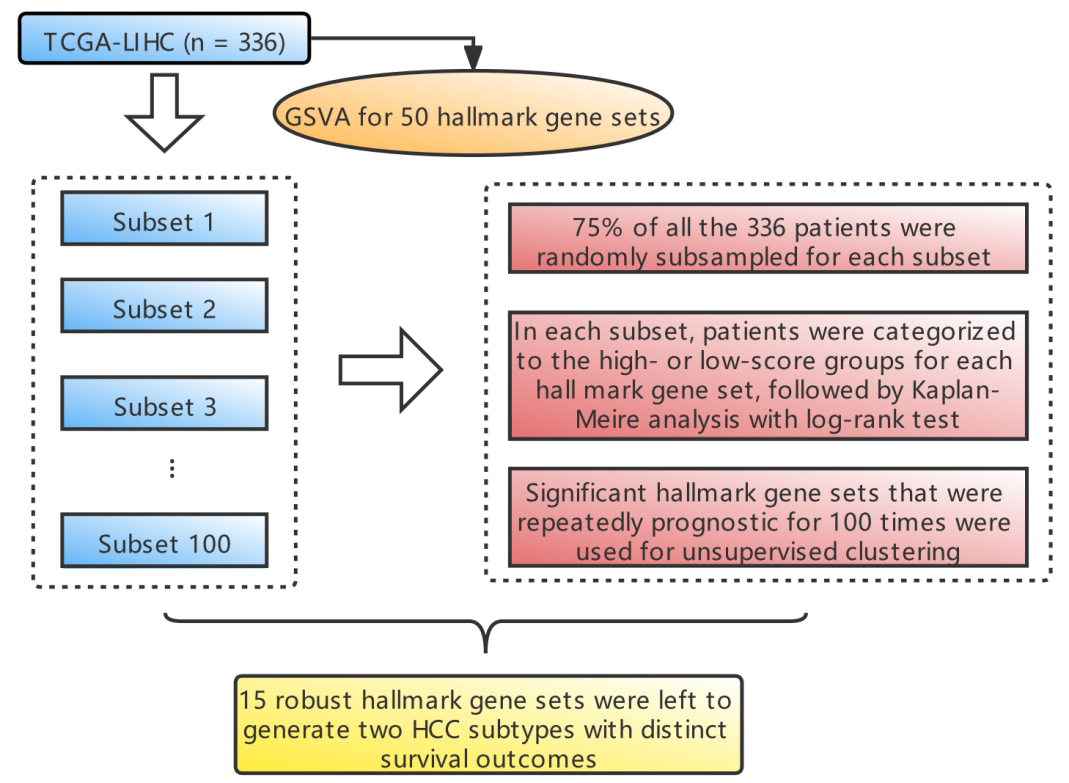


**Supplementary Figure 1 Screening of the robust prognostic hallmark gene sets with the “multi-split” strategy based on all 336 patients from the whole TCGA cohort.**

.


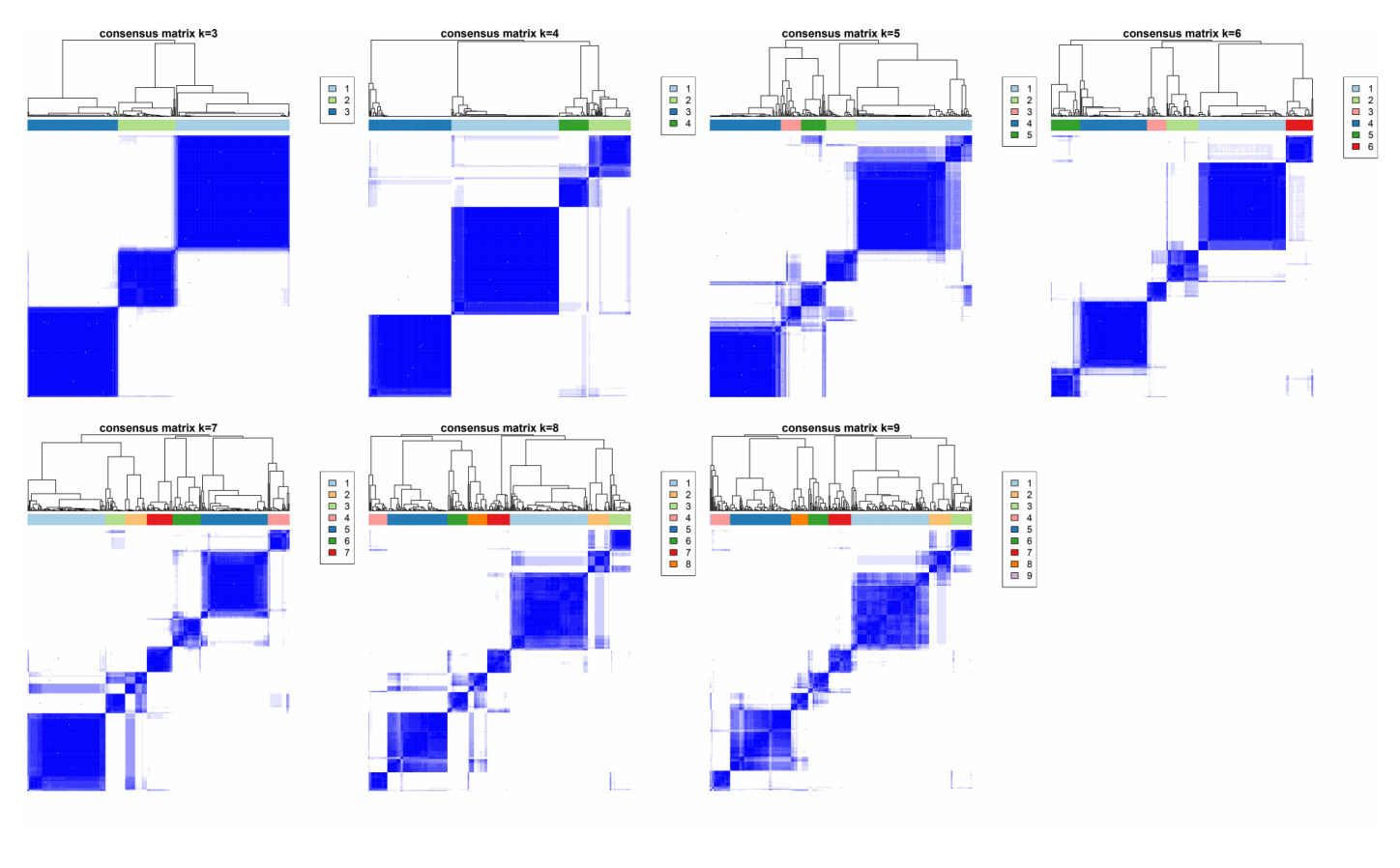


**Supplementary Figure 2 Unsupervised clustering of the 15 hallmark gene sets and consensus matrix heatmaps for k = 3-9.**


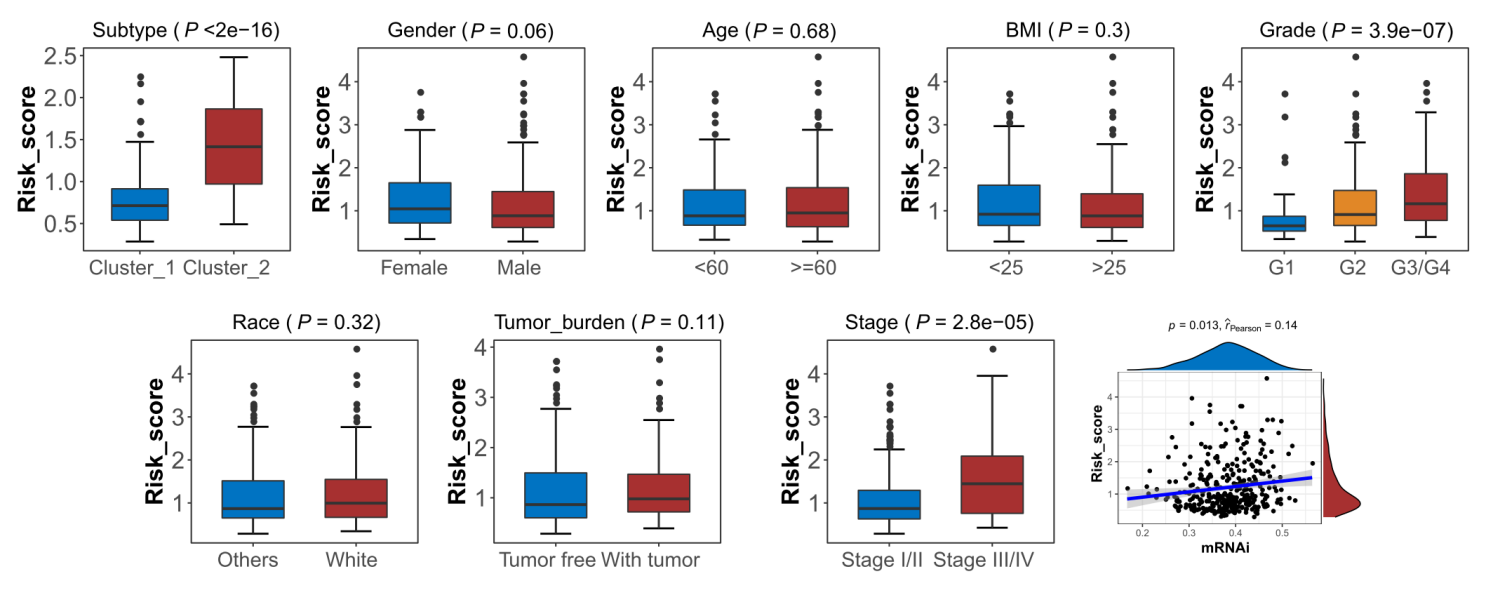


**Supplementary Figure 3 Boxplots and scatter plots showing the correlationszuo of HGSIS risk score and clinicopathologigal parameters.**


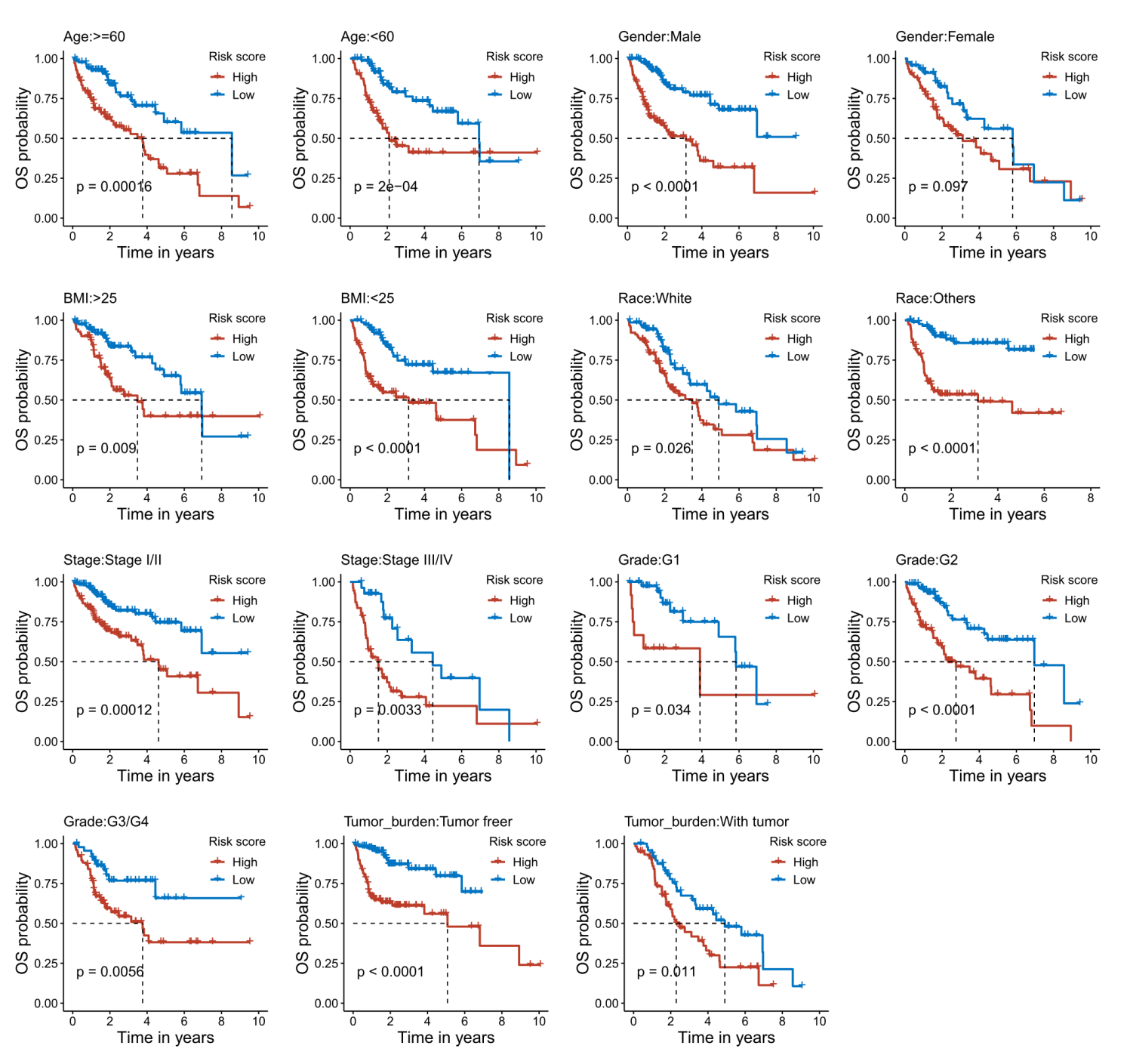


**Supplementary Figure 4 Kaplan-Meier curve demonstrates the prognostic value of HGSIS based on the subgroups of selected clinicopathological traits.**


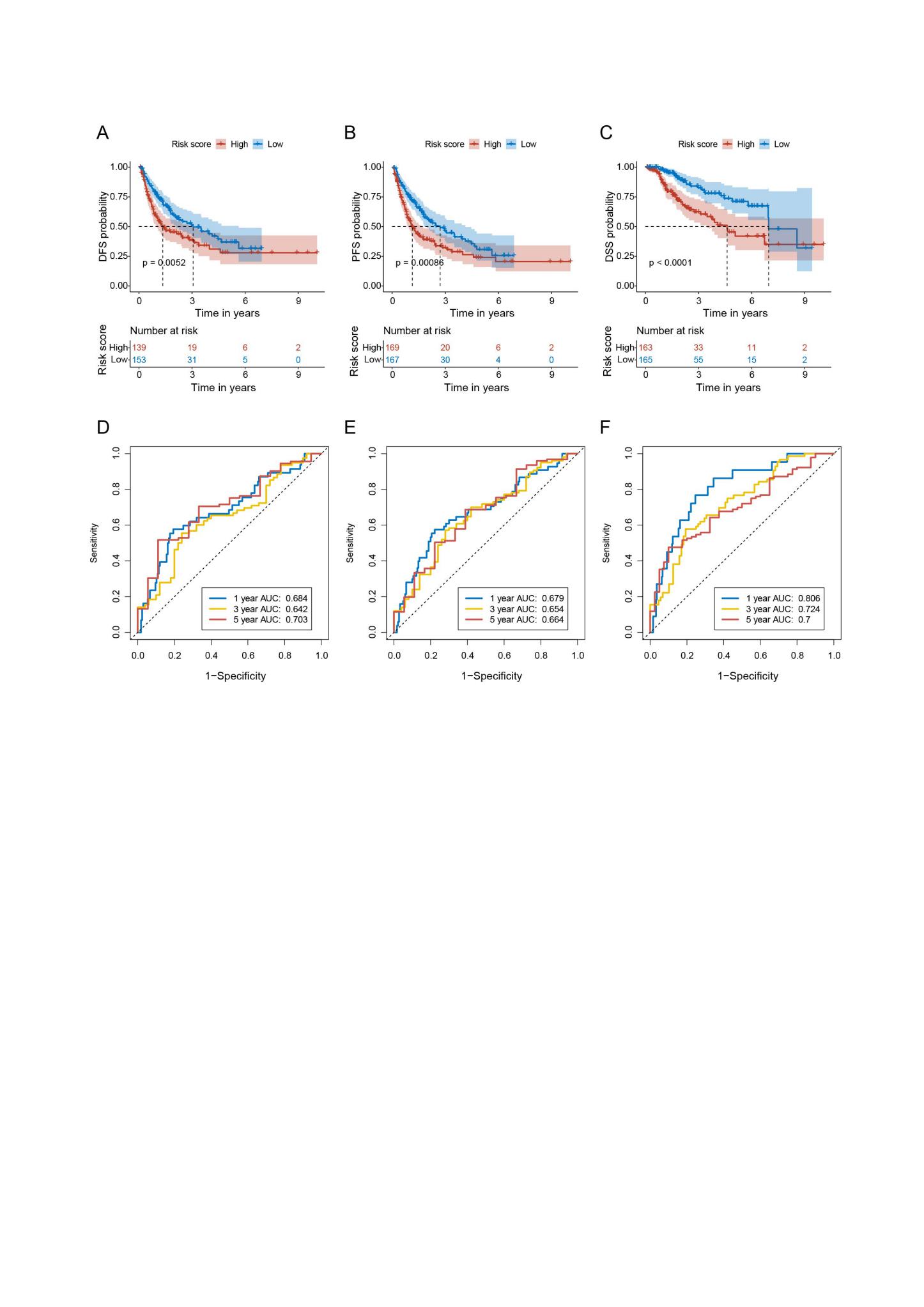


**Supplementary Figure 5 HGSIS could predict the DFS, PFS, and DSS of HCC patients effectively. (A-C)** Kaplan-Meier curves of HGSIS risk groups for the DFS, PFS, and DSS of HCC patients. **(D-F)** Time-dependent ROC plots of HGSIS risk score to evaluate the predictive accuracy of DFS, PFS, and DSS of HCC patients. PFI, progression-free survival; DFI, disease-free survival; DSS, disease-specific survival.


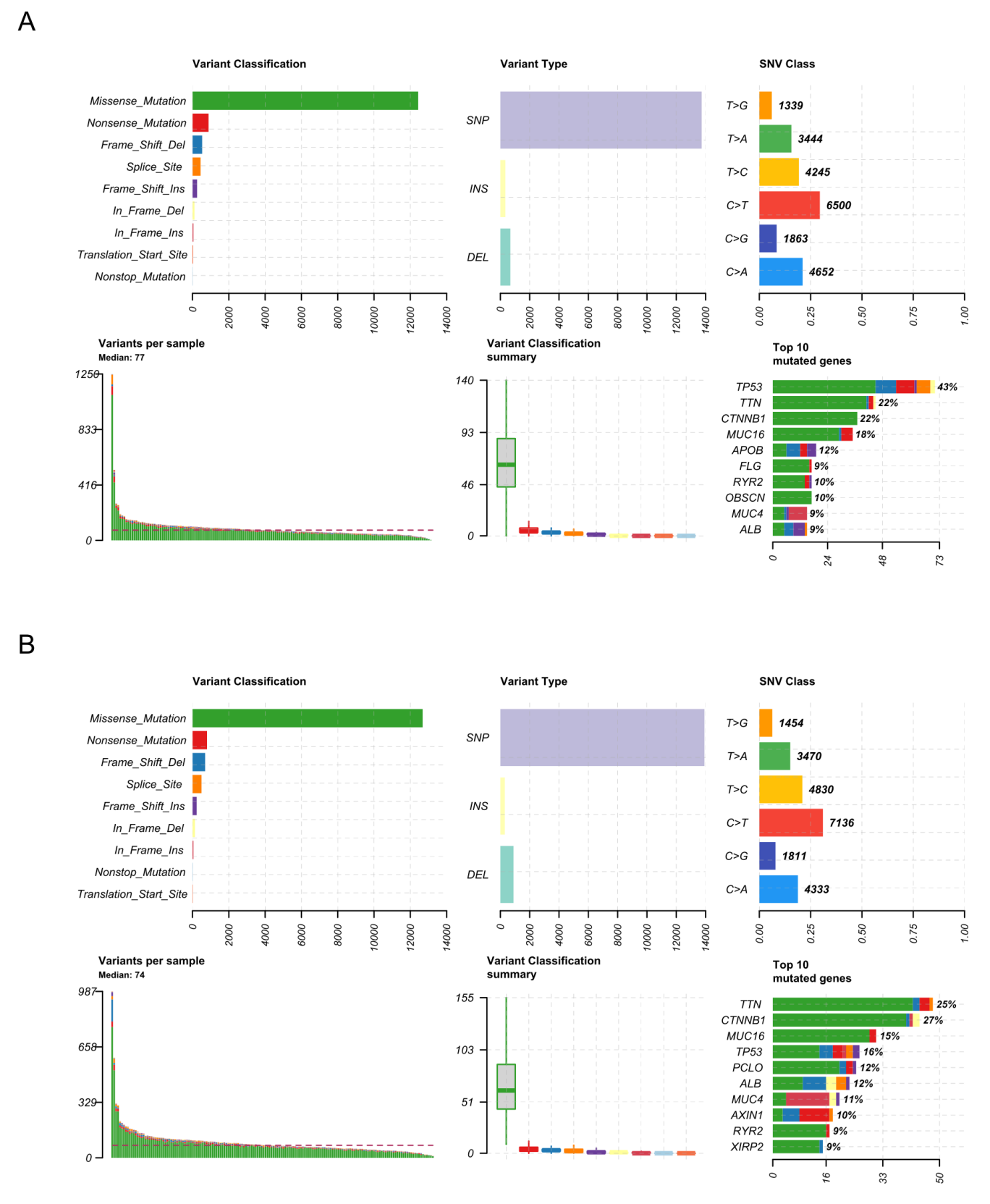


**Supplementary Figure 6 Summary of the mutation information in the high- (upper) and low- (bottom) risk groups with statistical calculations.**


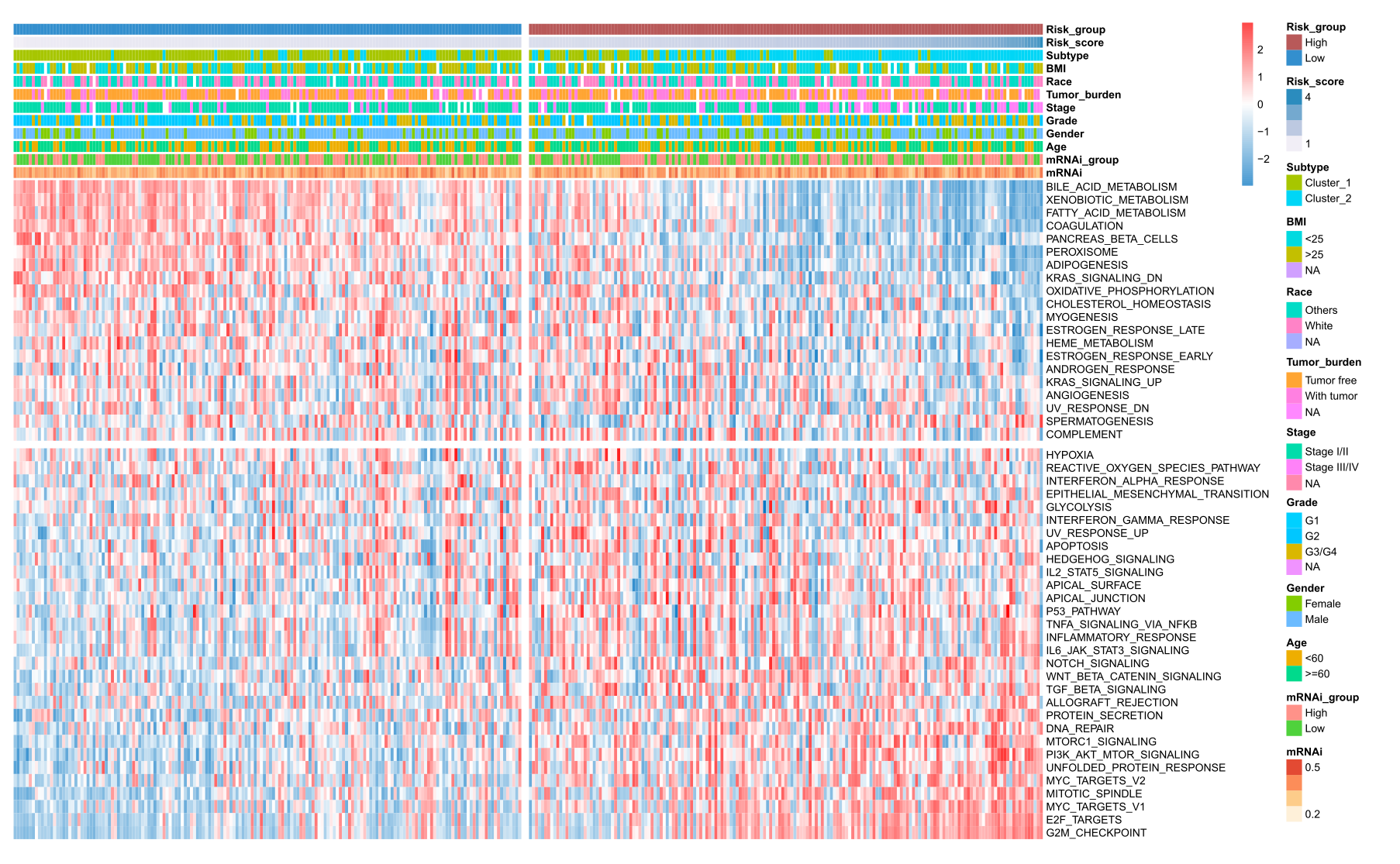


**Supplementary Figure 7 landscape of 50 hallmark gene sets annotated with HGSIS risk groups, HCC subtypes, and clinicopathological characteristics.**


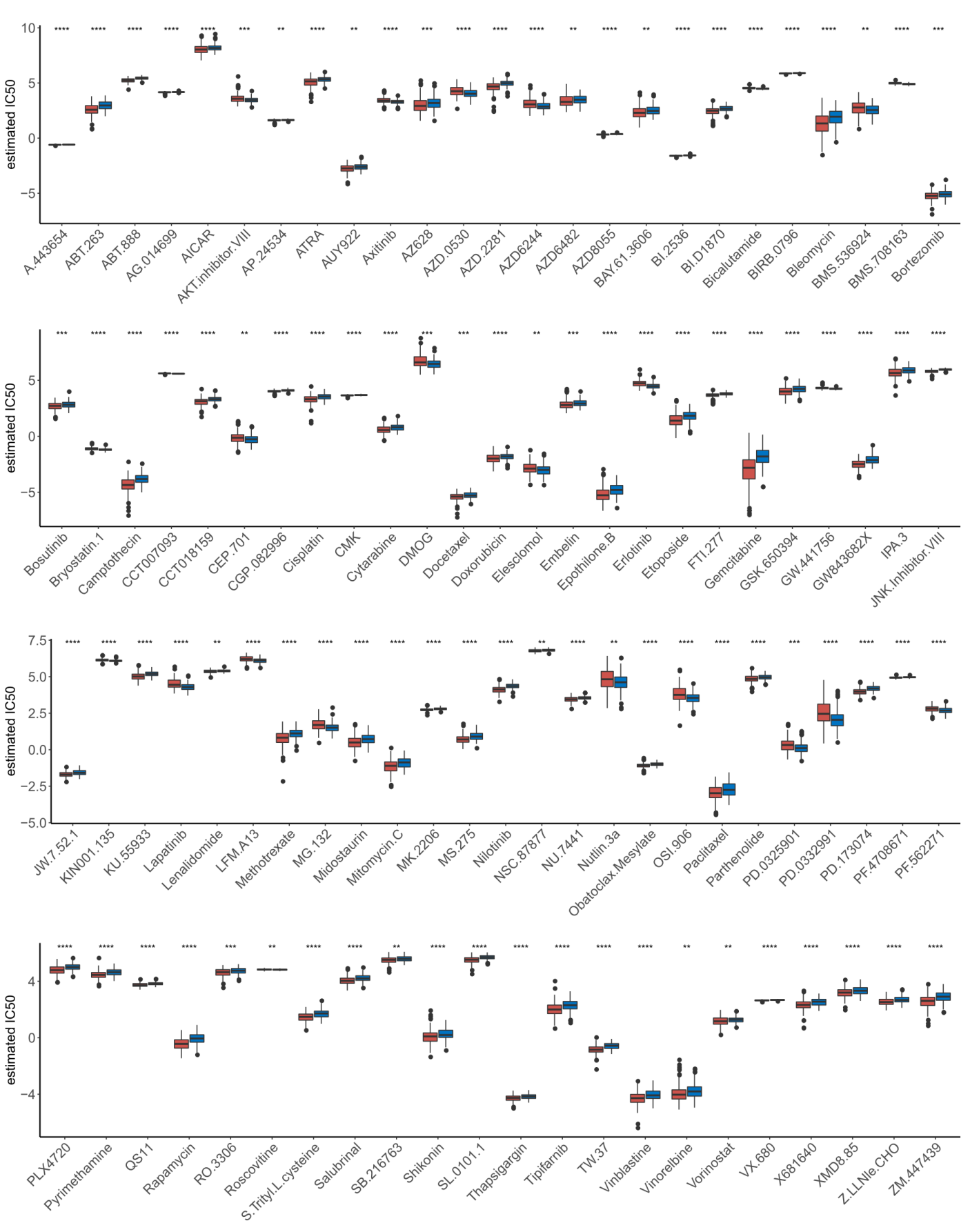


**Supplementary Figure 8 Boxplots illustrating the differential sensitivities of 138 targeted/chemo-therapeutic drugs.**


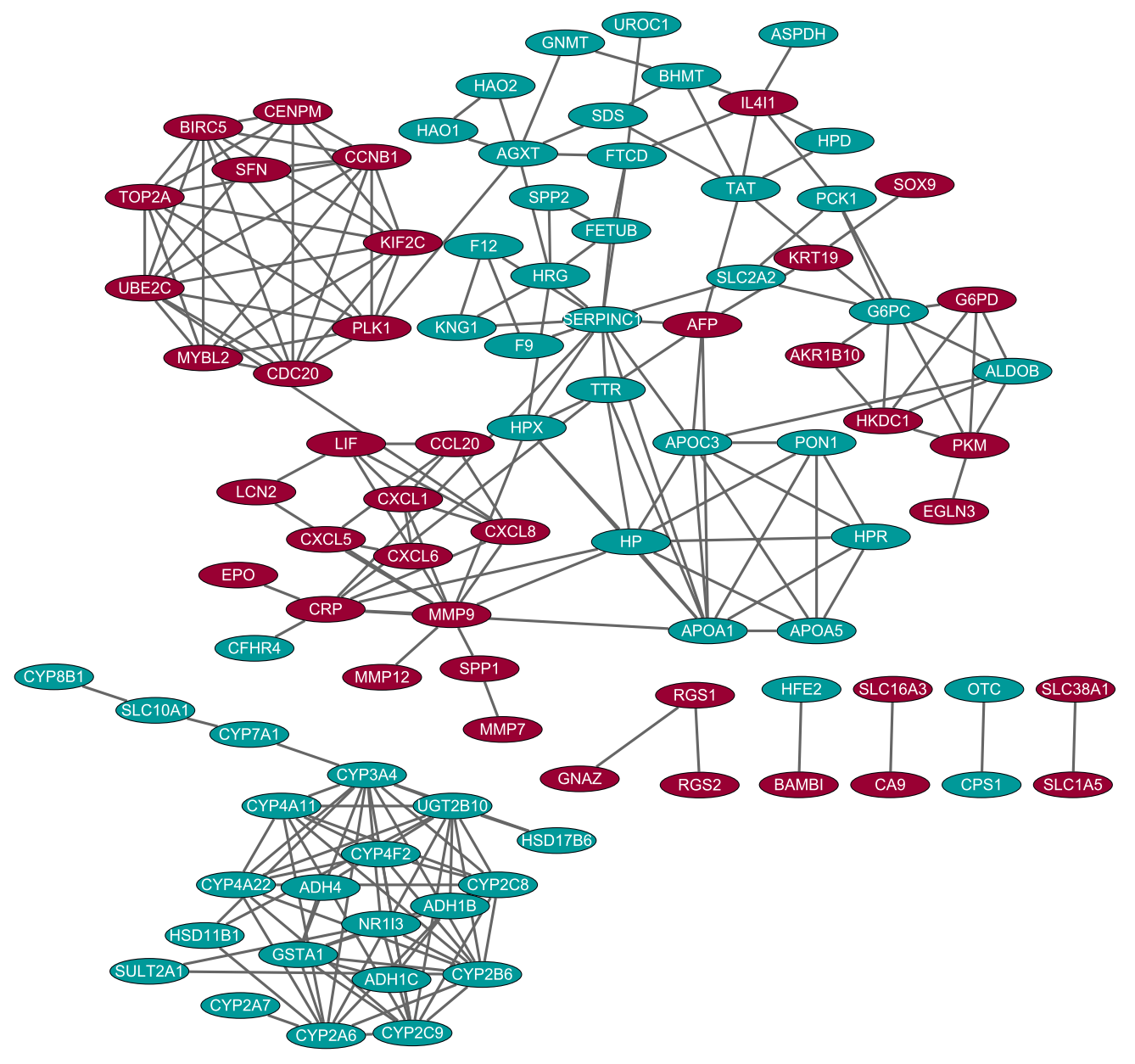


**Supplementary Figure 9 PPI network of DEGs concerning HGSIS. The red nodes indicate the upregulated genes, and the green nodes indicate the downregulated genes.**


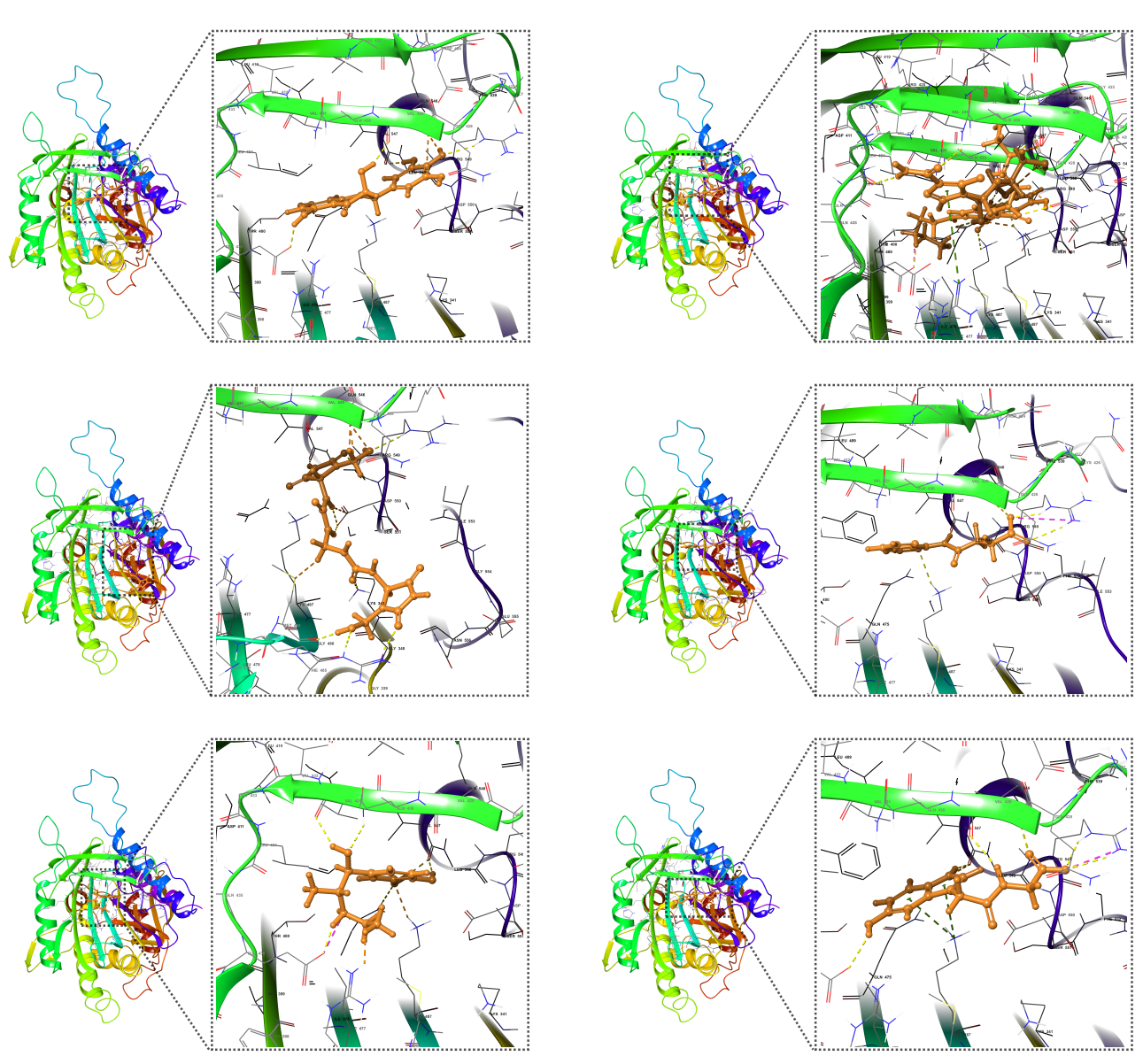


**Supplementary Figure 10 Structures and docking models of the leftover six small compounds (trans-3,3',4',5,5',7-hexahydroxyflavanone, mitoxantrone, imidurea, succinoadenin, dioxethedrin, and 5-hydroxyindoleacetylglycine) and the active site of KIF2C.**
